# Supplementary material for: Identifying the Bundle/Care Development Process in Clinical Risk Management: A Systematic Review
Source: Healthcare (Basel). 2024 Nov 11;12(22):2242. doi: 10.3390/healthcare12222242 (PMC11593500; doi:10.3390/healthcare12222242)
Supplement: Supplementary file 1 [file healthcare-12-02242-s001.zip › Multimedia File S1 (Search strategy).pdf]

Multimedia File S1 (Search strategy)

Search summary

List each resource searched here, for example:

Database Name	Platform	Date of Search	# of results
Agency for Health Care Research (AHRQ)	https://search.ahrq.gov/search?q=%22care+bundles%22	18/09/2023	1
Istitute for Healthcare Improvement (IHI)	https://www.ihi.org/sites/search/pages/results.aspx?k=care+bundle#k=care%20bundle#s=21	18/09/2023	5
PUBMED	https://pubmed.ncbi.nlm.nih.gov/?otool=iitbisamlib	17/09/2023	451
CINHAL	https://web.p.ebscohost.com/ehost/search/advanced?vid=1&sid=5cd8f541-ff4f-4a3a-a944-f10e761718aa%40redis	17/09/2023	328
EMBASE	https://www.embase.com/#advancedSearch/default	17/09/2023	587

Total Records = 1372

PubMed

Date of Search: 17/09/2023

Number of results: 451

Citation Alert Account Info:

#	Search string	# of results
1	"patient care bundle*"[Title/Abstract] OR "bundle*"[Title/Abstract] OR "care bundle*"[Title/Abstract] OR "evidence-based care bundle"[Title/Abstract] OR "evidence-based care bundle"[Title/Abstract] OR "best practice bundle*"[Title/Abstract] OR "care package*"[Title/Abstract] OR "care pathway*"[Title/Abstract] OR "care intervention*"[Title/Abstract] OR "prevention bundle*"[Title/Abstract] OR "care checklist"[Title/Abstract] OR "Patient Care Bundles"[MeSH Terms]	94118
2	"Clinical Protocols"[MeSH Terms] OR "Evidence-Based Practice"[MeSH Terms] OR "evidence-based"[Title/Abstract] OR "evidence-based"[Title/Abstract]	402444
3	“critical illness” OR “infection*” OR “medical error*” OR “sentinel event*” OR “near-miss” OR “near miss” OR “patient handoff” OR “adverse event*” OR “clinical risk” OR “risk management”)	2013080
4	1 AND #2 AND #3,,,"(""patient care bundle*""[Title/Abstract] OR ""bundle*""[Title/Abstract] OR ""care bundle*""[All Fields] OR ""evidence based care bundle*""[Title/Abstract] OR ""best practice bundle*""[Title/Abstract] OR ""care package*""[Title/Abstract] OR ""care pathway*""[Title/Abstract] OR ""care intervention*""[Title/Abstract] OR ""prevention bundle*""[Title/Abstract] OR ""care checklist""[Title/Abstract] OR ""Patient Care Bundles""[MeSH Terms]) AND (""clinical Protocols""[MeSH Terms] OR ""Evidence-Based Practice""[MeSH Terms] OR ""evidence-based""[Title/Abstract] OR ""clinical Protocols""[MeSH Terms] OR ""Evidence-Based Practice""[MeSH Terms] OR ""evidence-based""[Title/Abstract]) AND (""critical illness""[Title/Abstract] OR ""infection*""[Title/Abstract] OR ""medical error*""[Title/Abstract] OR ""sentinel event*""[Title/Abstract] OR ""near-miss""[Title/Abstract] OR ""near-miss""[Title/Abstract] OR ""patient handoff""[Title/Abstract] OR ""adverse event*""[Title/Abstract] OR ""clinical risk""[Title/Abstract] OR ""risk management""[Title/Abstract])"	531

5	#1 AND #2 AND #3	457
6	AND ((humans[Filter]) AND (2005/1/1:2023/9/17[pdat]))	451

CINHAL

Date of Search: 17/09/2023

Number of results: 328

Citation Alert Account Info:

#	**Search string**	**# of results**
1	TI "evidence-based care bundle" OR AB "evidence-based care bundle" OR TI "evidence based care bundle" OR AB "evidence based care bundle" OR TI '"best practice bundle'" OR AB '"best practice bundle'" OR TI "care pathway" OR AB "care pathway" OR TI "care package" OR AB "care package" OR TI "care intervention" OR AB "care intervention" OR TI "prevention bundle" OR AB "prevention bundle" OR TI "care checklist" OR AB "care checklist" OR TI "patient care bundle" OR AB "patient care bundle" OR TI "care bundle" OR AB "care bundle" OR TI "bundle" OR AB "bundle" OR TI "healthcare bundle" OR AB "healthcare bundle" OR TI "healthcare bundle" OR AB "healthcare bundle"	15457
2	MH protocols OR TI "protocols" OR TI "Evidence-Based Practice" OR AB "Evidence-Based Practice" OR TI "Evidence-Based" OR AB "Evidence-Based" OR TI "Evidence Based" OR AB "Evidence Based" OR AB "protocol*"	233675
3	TI "risk management" OR AB "risk management" OR TI "clinical risk" OR AB "clinical risk" OR AB "critical illness" OR TI "critical illness" OR AB "infection*" OR TI "infection*" OR AB "medical error" OR TI "medical error" OR AB "sentinel event" OR TI "sentinel event" OR TI "near miss" OR AB "near miss" OR TI "patient handoff" OR AB "patient handoff" OR TI "adverse event" OR AB "adverse event"	307675
4	#1 AND #2 and #3	347
5	AND (2005/1/1:2023/9/17[pdat]))	328

EMBASE

Date of Search: 17/09/2023

Number of results: 587

Citation Alert Account Info:

#	Search string	# of results
1	'evidence-based care bundle':ab,ti OR 'evidence based care bundle':ab,ti OR 'best practice bundle':ab,ti OR 'care package':ab,ti OR 'care pathway':ab,ti OR 'care intervention':ab,ti OR 'prevention bundle':ab,ti OR 'care checklist':ab,ti OR 'patient care bundle'/exp OR 'patient care bundle' OR 'care bundle'/exp OR bundle OR 'care bundle' OR 'healthcare bundle'/exp OR 'healthcare bundle'	97915
2	'clinical protocols'/exp OR 'evidence-based practice'/exp OR 'clinical protocols':ab,ti OR 'evidence-based practice':ab,ti	1929026
3	'critical illness':ab,ti OR 'infection':ab,ti OR 'medical error':ab,ti OR 'sentinel event':ab,ti OR 'near miss':ab,ti OR 'patient handoff':ab,ti OR 'adverse event':ab,ti OR 'clinical risk':ab,ti OR 'risk management':ab,ti	1892679
4	#1 AND #2 AND #3	651
5	AND [humans]/lim AND [2005-2023]/py	587

Agency for Health Care Research (AHRQ)

Date of Search: 18/09/2023

Number of results: 1

Citation Alert Account Info:

#	**Search string**	**# of results**
1	"care bundle"	135
2	"care bundles"	1

Istitute for Healthcare Improvement (IHI)
Date of Search: 18/09/2023
Number of results: 5
Citation Alert Account Info:

#	Search string	# of results
1	"care bundle"	5
