# Supplementary material for: Identifying the Bundle/Care Development Process in Clinical Risk Management: A Systematic Review
Source: Healthcare (Basel). 2024 Nov 11;12(22):2242. doi: 10.3390/healthcare12222242 (PMC11593500; doi:10.3390/healthcare12222242)
Supplement: Supplementary file 1 [file healthcare-12-02242-s001.zip › Multimedia File S2 (List of excluded studies).pdf]

Multimedia File S2 (List of excluded studies)

List of studies read in full text and excluded (with reasons for exclusion)

Studies	Reasons for exclusion
Assessment of knowledge and implementation practices of the ventilator acquired pneumonia (VAP) bundle in the intensive care unit of a private hospital. Antimicrob Resist Infect Control. 2021 nov 12;10(1):161. doi: 10.1186/s13756-021-01027-1. PMID: 34772458; PMCID:PMC8587499	Bundles that did not declare the stage of development
Proposed checklist of hospital interventions to decrease the incidence of healthcare-associated clostridium difficile infection	Bundles that did not declare the stage of development
Evaluation of the feasibility of the FAST-M maternal sepsis intervention in Pakistan: a protocol	Bundles that did not declare the stage of development
Decreasing ventilator-associated pneumonia in adult intensive care units using the Institute for Healthcare Improvement bundle. Am J Infect Control. 2010 Sep;38(7):552-6. doi: 10.1016/j.ajic.2010.01.008. Epub 2010 Apr 18. PMID: 20400203	Bundles that did not declare the stage of development
Preventive strategies for the reduction of central line-associated bloodstream infections in adult intensive care units: A systematic review	Bundles that did not declare the stage of development
Prevention of Ventilator-Associated Pneumonia: The Multimodal Approach of the Spanish ICU "Pneumonia Zero" Program. Crit Care Med. 2018 Feb;46(2):181-188. doi: 10.1097/CCM.0000000000002736. PMID: 29023261; PMCID: PMC5770104	Bundles that did not declare the stage of development
National survey of practices to prevent healthcare-associated infections in Thailand: The role of safety culture and collaboratives	Bundles that did not declare the stage of development
Implementing best practice strategies to prevent infection in the ICU. Crit Care Nurs Clin north Am. 2006 Dec;18(4):441-52. doi: 10.1016/j.ccell.2006.08.003. PMID: 17118299	Bundles that did not declare the stage of development
Does preventive care bundle have an impact on surgical site infections following spine surgery? An analysis of 9607 patients	Bundles that did not declare the stage of development
Adapting the ABCDEF Bundle to Meet the Needs of Patients Requiring Prolonged Mechanical Ventilation in the Long-Term Acute Care Hospital Setting: Historical Perspectives and Practical Implications. Semin Respir Crit Care Med. 2016 Feb;37(1):119-35. doi:10.1055/s-0035-1570361. Epub 2016 Jan 28. PMID: 26820279	Bundles that did not declare the stage of development
Implementing the awakening and breathing coordination, delirium monitoring/management, and early exercise/mobility bundle into everyday care: opportunities, challenges, and lessons learned for implementing the ICU Pain, Agitation, and Delirium Guidelines. Crit Care Med. 2013 Sep;41(9 Suppl 1):S116-27.doi: 10.1097/CCM.0b013e3182a17064. PMID: 23989089; PMCID: PMC4096994	Bundles that did not declare the stage of development
Reducing Clostridium difficile in the Inpatient Setting: A Systematic Review of the Adherence to and Effectiveness of C. difficile Prevention Bundles	Bundles that did not declare the stage of development
VAP free for 1000 days, it can be done. Crit Care Nurs Q. 2013 Oct-Dec;36(4):421-4. doi: 10.1097/CNQ.0b013e3182a1eb01. PMID: 24002432	Bundles that did not declare the stage of development
Collaborative cohort study of an intervention to reduce ventilator-associated pneumonia in the intensive care unit	Bundles that did not declare the stage of development
The 'bundle' approach to reduce the surgical site infection rate	Bundles that did not declare the stage of development

Studies	Reasons for exclusion
How to minimize central line–associated bloodstream infections in a neonatal intensive care unit: a quality improvement intervention based on a retrospective analysis and the adoption of an evidence-based bundle	Bundles that did not declare the stage of development
Ventilator-associated pneumonia in the pediatric intensive care unit: characterizing the problem and implementing a sustainable solution. J Pediatr. 2009 Apr;154(4):582-587.e2. doi: 10.1016/j.jpeds.2008.10.019. Epub 2008 Dec 3. PMID: 19054530	Bundles that did not declare the stage of development
Adherence to ventilator-associated pneumonia bundle and incidence of ventilator-associated pneumonia in the surgical intensive care unit. Arch Surg. 2010 May;145(5):465-70. doi: 10.1001/archsurg.2010.69. PMID: 20479345	Bundles that did not declare the stage of development
Prevention of central line-associated bloodstream infections through quality improvement interventions: A systematic review and meta-analysis	Bundles that did not declare the stage of development
Impact of quality improvement strategies for the prevention of central line-associated bloodstream infections: A systematic review and meta-analysis	Bundles that did not declare the stage of development
Reducing surgical site infections post-caesarean section in an Australian hospital, using a bundled care approach. Infect Dis Health. 2020 Aug;25(3):158-167. doi: 10.1016/j.idh.2020.01.006. Epub 2020 Mar 9. PMID: 32160964	Bundles that did not declare the stage of development
Implementation of a Wisconsin Division of Public Health Surgical Site Infection Prevention Champion Initiative. AORN J. 2018 May;107(5):570-578. doi: 10.1002/aorn.12123. PMID: 29708620	Bundles that did not declare the stage of development
A bundle of care to reduce colorectal surgical infections: an Australian experience. J Hosp Infect. 2011 Aug;78(4):297-301. doi:10.1016/j.jhin.2011.03.029. Epub 2011 Jun 12. PMID: 21664720	Bundles that did not declare the stage of development
The safe motherhood initiative: The development and implementation of standardized obstetric care bundles in New York. Semin Perinatol. 2016 Mar;40(2):124-31. doi: 10.1053/j.semperi.2015.11.019. Epub 2016 Jan 21. PMID: 26804380	Bundles that did not declare the stage of development
Adherence to the central line bundle in intensive care: An integrative review. Am J Infect Control. 2021 Jul;49(7):937-956. doi: 10.1016/j.ajic.2020.11.014. Epub 2020 nov 19. PMID: 33221472	Bundles that did not declare the stage of development
An in-depth look at ventilator-associated pneumonia in trauma patients and efforts to increase bundle compliance, education and documentation in a surgical trauma critical care unit. Am J Infect Control. 2022 Dec;50(12):1333-1338. doi: 10.1016/j.ajic.2022.01.029. Epub 2022 Feb 4. PMID:35131347	Bundles that did not declare the stage of development
An evidence-based catheter bundle alters central venous catheter strategy in newborn infants	Bundles that did not declare the stage of development
HANDS: standardised intravascular practice based on evidence. Br J Nurs. 2012 Jul 26-Aug 8;21(14):S4, S6, S8-11. doi: 10.12968/bjon.2012.21.Sup14.S4. PMID: 23252175	Bundles that did not declare the stage of development
Bundling: the KISS method for preventing HAIs	Bundles that did not declare the stage of development
An evidence-based bundle improves the quality of care and outcomes of patients with candidaemia	Bundles that did not declare the stage of development
An evidence-based bundle improves the quality of care and outcomes of patients with candidaemia. J Antimicrob Chemother. 2020 Mar 1;75(3):730-737. doi: 10.1093/jac/dkz491. PMID:31819991	Bundles that did not declare the stage of development
Evidence-Based Bundles and Cesarean Delivery Surgical Site Infections: A Systematic Review and Meta-analysis. Obstet Gynecol. 2017 Oct;130(4):735-746. doi: 10.1097/AOG.0000000000002249. PMID: 28885421	Bundles that did not declare the stage of development
An Evidence-Based Approach To the Prevention of Catheter-Associated Urinary Tract Infections. Urol Nurs. 2014 Sep-Oct;34(5):238-45. PMID: 26298933	Bundles that did not declare the stage of development
Use of protocols in older intensive care unit patients: is standardization appropriate? AACN Adv Crit Care. 2011 Apr-Jun;22(2):150-60. doi: 10.1097/NCI.0b013e3182156cc7. PMID: 21521957	Bundles that did not declare the stage of development
Impact of Surgical Care Bundle on Surgical Site Infection after Non-Reconstructive Breast Cancer Surgery: A Single-Centre Retrospective Comparative Cohort Study	Bundles that did not declare the stage of development
Guidelines for severe infections: Are they useful?	Bundles that did not declare the stage of development

Studies	Reasons for exclusion
Temporary hemodialysis catheters: recent advances. Kidney Int. 2014 nov;86(5):888-95. doi: 10.1038/ki.2014.162. Epub 2014 May 7. PMID: 24805107; PMCID: PMC4220490	Bundles that did not declare the stage of development
Reduction in catheter-associated urinary tract infections by bundling interventions. Int J Qual Health Care. 2013 Feb;25(1):43-9. doi: 10.1093/intqhc/mzs077. Epub 2012 Dec 6. PMID: 23220761	Bundles that did not declare the stage of development
PreciSSIon: a collaborative initiative to reduce surgical site infections after elective colorectal surgery. J Hosp Infect. 2022 Dec;130:131-137. doi: 10.1016/j.jhin.2022.08.012. Epub 2022 Sep 7. PMID: 36087804	Bundles that did not declare the stage of development
Preventing ventilator-associated pneumonia in children: an evidence-based protocol. Crit Care Nurse. 2013 Jun;33(3):21-9; quiz 30. doi:10.4037/ccn2013204. PMID: 23727849	Bundles that did not declare the stage of development
Systematic intervention to reduce central line-associated bloodstream infection rates in a pediatric cardiac intensive care unit. Pediatrics. 2008 May;121(5):915-23. doi: 10.1542/peds.2007-1577. PMID: 18450894	Bundles that did not declare the stage of development
Minimizing complications associated with percutaneous central venous catheter placement in children: recent advances. Pediatr Crit Care Med. 2013 Mar;14(3):273-83. doi: 10.1097/PCC.0b013e318272009b.PMID: 23392365	Bundles that did not declare the stage of development
Effects of a predictive preventive model for prevention of Clostridium difficile infection in patients in intensive care units. Am J Infect Control. 2016 Apr 1;44(4):421-4. doi:10.1016/j.ajic.2015.11.010. Epub 2016 Jan 5. PMID: 26775936	Bundles that did not declare the stage of development
Using the knowledge translation framework to change practical care of central catheters in a Brazilian neonatal unit	Bundles that did not declare the stage of development
Implementation of a Ventilator-Associated Pneumonia Prevention Bundle in a Single PICU. Pediatr Crit Care Med. 2016 May;17(5):451-6. doi: 10.1097/PCC.0000000000000714. PMID: 27043995	Bundles that did not declare the stage of development
Enhanced PeriOperative Care and Health protection programme for the prevention of surgical site infections after elective abdominal surgery (EPOCH): study protocol of a randomised controlled, multicentre, superiority trial. BMJ Open. 2020 May 25;10(5):e038196. doi: 10.1136/bmjopen-2020-038196. PMID: 32457082; PMCID: PMC7252990	Bundles that did not declare the stage of development
Failure to implement evidence-based clinical guidelines for sepsis at the ED. Am J Emerg Med. 2006 Sep;24(5):553-9. doi: 10.1016/j.ajem.2006.01.012. PMID: 16938593	Bundles that did not declare the stage of development
Introduction of a care bundle effectively and sustainably reduces patient-reported surgical site infection in patients undergoing colorectal surgery. J Hosp Infect. 2020 Jun;105(2):156-161. doi: 10.1016/j.jhin.2020.04.013. Epub 2020 Apr 11. PMID:32289384	Bundles that did not declare the stage of development
Impact of Bundled Intervention on Outcomes of Patients Undergoing Clean Orthopedic Surgeries With Hardware Implants: Small Prospective Randomized Controlled Trial	Bundles that did not declare the stage of development
Impact of Bundled Intervention on Outcomes of Patients Undergoing Clean Orthopedic Surgeries With Hardware Implants: Small Prospective Randomized Controlled Trial. Surg Infect (Larchmt). 2023 Aug;24(6):566-574. doi: 10.1089/sur.2023.119. PMID: 37526644	Bundles that did not declare the stage of development
Surgical site infection prevention bundle in elective colorectal surgery. J Hosp Infect. 2022 Apr;122:162-167. doi: 10.1016/j.jhin.2022.01.023. Epub 2022 Feb 10. PMID:35151765	Bundles that did not declare the stage of development
Prevention of Orthopedic Prosthetic Infections Using Evidence-Based Surgical Site Infection Care Bundles: A Narrative Review. Surg Infect (Larchmt). 2022 Sep;23(7):645-655. doi: 10.1089/sur.2022.146. Epub 2022 Aug 4. PMID: 35925775	Bundles that did not declare the stage of development
Assessment of risk and economic burden of surgical site infection (SSI) posthysterectomy using a U.S. longitudinal database. Surgery. 2022 May;171(5):1320-1330. doi: 10.1016/j.surg.2021.11.034. Epub 2021 Dec 29. PMID: 34973811	Bundles that did not declare the stage of development
The ABCDEF Bundle: Science and Philosophy of How ICU Liberation Serves Patients and Families. Crit Care Med. 2017 Feb;45(2):321-330. doi: 10.1097/CCM.0000000000002175. PMID: 28098628; PMCID: PMC583012	Bundles that did not declare the stage of development
The ABCDEF bundle: Science and philosophy of how ICU liberation serves patients and families	Bundles that did not declare the stage of development
Gruppo Aperto di Studio sugli Accessi Venosi Centrali a Lungo Termine. Il protocollo 'ISP' (Inserzione Sicura dei PICC): un "bundle" di otto raccomandazioni per minimizzare le complicanze legate all'impianto dei cateteri centrali ad inserimento periferico (PICC) [The ISP (Safe Insertion of PICCs) protocol: a bundle of 8 recommendations to minimize the complications related to the peripherally inserted central venous catheters (PICC)]. Assist Inferm Ric. 2014 Apr-Jun;33(2):82-9. Italian. doi: 10.1702/1539.16813. PMID: 25002059	Bundles that did not declare the stage of development

Studies	Reasons for exclusion
Effectiveness of a care bundle to reduce central line-associated bloodstream infections. Med J Aust. 2015 Mar 16;202(5):247-50. doi: 10.5694/mja14.01644. PMID: 25758694	Bundles that did not declare the stage of development
Beyond the bundle--journey of a tertiary care medical intensive care unit to zero central line-associated bloodstream infections. Crit Care. 2013 Mar 4;17(2):R41. doi: 10.1186/cc12551. PMID: 23497591; PMCID: PMC3733431.	Bundles that did not declare the stage of development
Beyond the bundle - journey of a tertiary care medical intensive care unit to zero central line-associated bloodstream infections	Bundles that did not declare the stage of development
Implementation of an organ space infection prevention bundle reduces the rate of organ space infection after elective colorectal surgery	Bundles that did not declare the stage of development
Implementation of an organ space infection prevention bundle reduces the rate of organ space infection after elective colorectal surgery. BMJ Open Qual. 2021 May;10(2):e001278. doi: 10.1136/bmjoq-2020-001278. PMID: 33941539; PMCID: PMC8098924	Bundles that did not declare the stage of development
Surgical site infection in bariatric surgery: results of a care bundle. Rev Col Bras Cir. 2019 Sep 9;46(4):e2252. Portuguese, English. doi: 10.1590/0100-6991e-20192252. PMID: 31508737	Bundles that did not declare the stage of development
Enquête multicentrique sur l'application des recommandations de prévention des pneumopathies acquises sous ventilation mécanique en réanimation [Multicenter survey on ventilator- associated pneumonia prevention in intensive care]. Ann Fr Anesth Reanim. 2013 Dec;32(12):833-7. French. doi: 10.1016/j.annfar.2013.07.822. Epub 2013 nov 1.PMID: 24184168	Bundles that did not declare the stage of development
Infection prevention for extremely low birth weight infants in the NICU. Semin Fetal Neonatal Med. 2022 Jun;27(3):101345.doi: 10.1016/j.siny.2022.101345. Epub 2022 Apr 13. PMID: 35550785; PMCID:<br>PMC9006400	Bundles that did not declare the stage of development
Preventing Central Line Blood Stream Infections in Critical Care Patients. Crit Care Nurs Clin north Am. 2021 Dec;33(4):419-429. doi: 10.1016/j.cnc.2021.08.001. Epub 2021 Oct 9. PMID: 34742498	Bundles that did not declare the stage of development
The evaluation of the implementation of the vascular preventative bundle and development of suggested interventions for improvement and sustainability. J Vasc Nurs. 2018 Mar;36(1):8-11. doi: 10.1016/j.jvn.2017.09.002. Epub 2017 nov 15. PMID:29452631	Bundles that did not declare the stage of development
What is the evidence behind recommendations for infection control? Adv Exp Med Biol. 2011;719:163-9. doi:10.1007/978-1-4614-0204-6_14. PMID: 22125043	Bundles that did not declare the stage of development
The impact of compliance with 6-hour and 24-hour sepsis bundles on hospital mortality in patients with severe sepsis: a prospective observational study	Bundles that did not declare the stage of development
The impact of compliance with 6-hour and 24-hour sepsis bundles on hospital mortality in patients with severe sepsis: a prospective observational study. Crit Care. 2005;9(6):R764-70. doi: 10.1186/cc3909. Epub 2005 nov 11. PMID: 16356225; PMCID: PMC1414020	Bundles that did not declare the stage of development
Randomized controlled trial of care bundles with chlorhexidine dressing and advanced dressings to prevent catheter-related bloodstream infections in pediatric hematology-oncology patients	Bundles that did not declare the stage of development
Implementation of a multifaceted nurse-led intervention to reduce indwelling urinary catheter use in four Australian hospitals: A pre- and postintervention study. J Clin Nurs. 2020 Mar;29(5-6):872-886. doi: 10.1111/jocn.15142. Epub 2020 Jan 2. PMID: 31856344	Bundles that did not declare the stage of development
Does our bundle stack up! Innovative nurse-led changes for preventing catheter-associated urinary tract infection (CAUTI)	Bundles that did not declare the stage of development
An Effective Bundled Approach Reduces Surgical Site Infections in a High-Outlier Colorectal Unit	Bundles that did not declare the stage of development
Use of a Central Catheter Maintenance Bundle in Long-Term Acute Care Hospitals. Am J Crit Care. 2016 Mar;25(2):165-72. doi: 10.4037/ajcc2016894. PMID: 26932919	Bundles that did not declare the stage of development
Evaluating the effect of a neonatal care bundle for the prevention of intraventricular hemorrhage in preterm infants	Bundles that did not declare the stage of development
Bundle approach used to achieve zero central line-associated bloodstream infections in an adult coronary intensive care unit. BMJ Open Qual. 2021 Feb;10(1):e001200. doi: 10.1136/bmjoq-2020-001200. PMID: 33597274; PMCID: PMC7893645	Bundles that did not declare the stage of development

Studies	Reasons for exclusion
Reducing catheter-associated urinary tract infections in the cardiac intensive care unit with a coordinated strategy and nursing staff empowerment	Bundles that did not declare the stage of development
Researching effective approaches to cleaning in hospitals: protocol of the REACH study, a multi-site stepped-wedge randomised trial	Bundles that did not declare the stage of development
Hall L, Farrington A, Mitchell BG, Barnett AG, Halton K, Allen M, Page K, Researching effective approaches to cleaning in hospitals: protocol of the REACH study, a multi-site stepped-wedge randomised trial. Implement Sci.2016 Mar 24;11:44. doi: 10.1186/s13012-016-0406-6. PMID: 27009342; PMCID:PMC4806497	Bundles that did not declare the stage of development
Effectiveness of a structured, framework-based approach to implementation: the Researching Effective Approaches to Cleaning in Hospitals (REACH) Trial. Antimicrob Resist Infect Control. 2020 Feb 18;9(1):35. doi: 10.1186/s13756-020-0694-0. PMID: 32070419; PMCID: PMC7029491	Bundles that did not declare the stage of development
Evidence-based clinical improvement for mechanically ventilated patients. Rehabil Nurs. 2005 Jul-Aug;30(4):160-5. doi: 10.1002/j.2048-7940.2005.tb00101.x. PMID: 15999861	Bundles that did not declare the stage of development
A multidisciplinary intervention to reduce central line-associated bloodstream infection in pediatrics and neonatal intensive care units. Pediatr Neonatol. 2022 Jan;63(1):71-77. doi: 10.1016/j.pedneo.2021.08.010. Epub 2021 Sep 23. PMID: 34620576	Bundles that did not declare the stage of development
Falls prevention in hospitals and mental health units: an extended evaluation of the FallSafe quality improvement project. Age Ageing. 2014 Jul;43(4):484-91. doi: 10.1093/ageing/aft190. Epub 2013 Dec 8. PMID: 24321841	Bundles that did not declare the stage of development
Evidence-Based Bundled Quality Improvement Intervention for Reducing Surgical Site Infection in Lower Extremity Vascular Bypass Procedures	Bundles that did not declare the stage of development
Evidence-Based Bundled Quality Improvement Intervention fr Reducing Surgical Site Infection in Lower Extremity Vascular Bypass Procedures. J Am Coll Surg. 2019 Jan;228(1):44-53. doi: 10.1016/j.jamcollsurg.2018.10.002. Epub 2018 Oct 22. PMID: 30359836; PMCID: PMC6557406	Bundles that did not declare the stage of development
Improving compliance with central venous catheter care bundles using electronic records. Nurs Crit Care. 2015 Jul;20(4):196-203. doi:10.1111/nicc.12186. Epub 2015 May 13. PMID: 25968111	Bundles that did not declare the stage of development
Catheter-associated urinary tract infection (CAUTI) prevention and nurses' checklist documentation of their indwelling catheter management practices.	Bundles that did not declare the stage of development
Reducing colorectal surgical site infections: a novel, resident-driven, quality initiative. Am J Surg. 2017 Jan;213(1):36-42. doi: 10.1016/j.amjsurg.2016.04.009. Epub 2016 Jun 14. PMID: 27427296	Bundles that did not declare the stage of development
Perioperative Bundle Decreases Postoperative Hepatic Surgery Infections. Ann Surg Oncol. 2015 Dec;22 Suppl 3:S1140-6. doi: 10.1245/s10434-015-4584-2. Epub 2015 May 14. PMID: 25971958	Bundles that did not declare the stage of development
Effective strategies for reducing the incidence of catheter-related bloodstream infections. Br J Nurs. 2014;23(14 Suppl):S4-10. PMID: 25373250	Bundles that did not declare the stage of development
Impact of a quality improvement intervention on the incidence of surgical site infection in patients undergoing colorectal surgery: Pre-test-post-test design. J Clin Nurs. 2023 Aug;32(15-16):4932-4946. doi: 10.1111/jocn.16690. Epub 2023 Mar 16. PMID:36924125	Bundles that did not declare the stage of development
Reduction and sustainability of cesarean section surgical site infection: An evidence-based, innovative, and multidisciplinary quality improvement intervention bundle program	Bundles that did not declare the stage of development
Reduction and sustainability of cesarean section surgical site infection: An evidence-based, innovative, and multidisciplinary quality improvement intervention bundle program. Am J Infect Control. 2016 nov 1;44(11):1315-1320. doi: 10.1016/j.ajic.2016.04.217. Epub 2016 Jun 14. PMID:27317407	Bundles that did not declare the stage of development
Effect of a patency bundle on central venous catheter complications among hospitalized adult patients: A best practice implementation project	Bundles that did not declare the stage of development
Effect of a patency bundle on central venous catheter complications among hospitalized adult patients: a best practice implementation project. JBI Database System Rev Implement Rep. 2018 Feb;16(2):565-586. doi: 10.11124/JBISRIR-2016-003340. PMID: 29419626	Bundles that did not declare the stage of development
How-to Guide:Prevent Central Line-Updated March 2012 Associated Bloodstream Infections (CLABSI)	Bundles that did not declare the stage of development

Studies	Reasons for exclusion
How-to Guide: Prevent Obstetrical Adverse Events	Bundles that did not declare the stage of development
How-to Guide: Prevent Ventilator-Associated Pneumonia	Bundles that did not declare the stage of development
ZAP-VAP: A Quality Improvement Initiative to Decrease Ventilator-Associated Pneumonia in the Neonatal Intensive Care Unit, 2012-2016	Bundles that did not declare the stage of development
Repeated multimodal supervision programs to reduce the central line-associated bloodstream infection rates in an Indian corporate hospital	Bundles that did not declare the stage of development
Prevention of central venous catheter-associated bloodstream infections in pediatric intensive care units: a performance improvement collaborative. Infect Control Hosp Epidemiol. 2009 Jul;30(7):645-51. doi: 10.1086/598341. PMID: 19496731	Bundles that did not declare the stage of development
Effect of central line bundle on central line-associated bloodstream infections in intensive care units	Bundles that did not declare the stage of development
Using Bundled Interventions to Reduce Surgical Site Infection After Major Gynecologic Cancer Surgery	Bundles that did not declare the stage of development
Using Bundled Interventions to Reduce Surgical Site Infection After Major Gynecologic Cancer Surgery. Obstet Gynecol. 2016 Jun;127(6):1135-1144. doi: 10.1097/AOG.0000000000001449. PMID: 27159744	Bundles that did not declare the stage of development
Challenges related to surgical site infection prevention—results after standardized bundle implementation	Bundles that did not declare the stage of development
Impact of nurse-led remote screening and prompting for evidence-based practices in the ICU	Bundles that did not declare the stage of development
Impact of an Antibiotic-specific Sepsis Bundle on Appropriate and Timely Antibiotic Administration for Severe Sepsis in the Emergency Department	Bundles that did not declare the stage of development
Surgical technical evidence review for gynecologic surgery conducted for the Agency for Healthcare Research and Quality Safety Program for Improving Surgical Care and Recovery. Am J Obstet Gynecol. 2018 Dec;219(6):563.e1-563.e19. doi:10.1016/j.ajog.2018.07.014. Epub 2018 Jul 19. PMID: 30031749	Bundles that did not declare the stage of development
Failure of central venous catheter insertion and care bundles in a high central line–associated bloodstream infection rate, high bed occupancy hospital	Bundles that did not declare the stage of development
Reducing Cesarean Delivery Surgical Site Infections: A Resident-Driven Quality Initiative. Obstet Gynecol. 2019 Feb;133(2):282-288. doi: 10.1097/AOG.0000000000003091. PMID: 30633146; PMCID: PMC6353635	Bundles that did not declare the stage of development
A bundle that includes active surveillance, contact precaution for carriers, and cefazolin-based antimicrobial prophylaxis prevents methicillin-resistant Staphylococcus aureus infections in clean orthopedic surgery	Bundles that did not declare the stage of development
Back to Basics: CLABSI Reduction through Implementation of an Oral Care and Hygiene Bundle. J Pediatr Oncol Nurs. 2019 Sep/Oct;36(5):321-326. doi: 10.1177/1043454219849583. Epub 2019 May 17.PMID: 31096826	Bundles that did not declare the stage of development
Prevention of ventilator-associated pneumonia in the intensive care unit: a review of the clinically relevant recent advancements. Indian J Med Res. 2014 Jun;139(6):814-21. PMID: 25109715; PMCID:PMC4164993	Bundles that did not declare the stage of development
The impact of implementing multifaceted interventions on the prevention of ventilator-associated pneumonia	Bundles that did not declare the stage of development
Implementing the comprehensive unit-based safety program model to improve the management of mechanically ventilated patients in Saudi Arabia	Bundles that did not declare the stage of development
Quality toolbox. Daily goals checklist -- a goal-directed method to eliminate nosocomial infection in the intensive care unit	Bundles that did not declare the stage of development
Daily goals checklist--a goal-directed method to eliminate nosocomial infection in the intensive care unit. J Healthc Qual. 2008 nov- Dec;30(6):13-7. doi: 10.1111/j.1945-1474.2008.tb01165.x. PMID: 19160870	Bundles that did not declare the stage of development

Studies	Reasons for exclusion
Improving the central venous access devices maintenance process to reduce associated infections in paediatrics: evaluation of a practical, multi-faceted quality-improvement initiative	Bundles that did not declare the stage of development
SMART approaches for reducing nosocomial infections in the ICU. Chest. 2008 Aug;134(2):447-456. doi: 10.1378/chest.08-0809. PMID: 18682466	Bundles that did not declare the stage of development
Ventilator-associated complications, including infection-related complications: the way forward. Crit Care Clin. 2013 Jan;29(1):33-50. doi: 10.1016/j.ccc.2012.10.004. Epub 2012 nov 6. PMID: 23182526	Bundles that did not declare the stage of development
Prevention of ventilator-associated pneumonia or ventilator- associated complications: a worthy, yet challenging, goal. Crit Care Med. 2012 Jan;40(1):271-7. doi: 10.1097/CCM.0b013e318232e41d. PMID: 21946659	Bundles that did not declare the stage of development
Reducing paediatric ventilator-associated pneumonia -- a South African challenge!	Bundles that did not declare the stage of development
The ventilator care bundle and its impact on ventilator-associated pneumonia: a review of the evidence.	Bundles that did not declare the stage of development
Evidence update on prevention of surgical site infection. Curr Opin Infect Dis. 2015 Apr;28(2):158-63. doi: 10.1097/QCO.0000000000000144. PMID: 25692267.	Bundles that did not declare the stage of development
Surgical site infection: poor compliance with guidelines and care bundles. Int Wound J. 2015 Jun;12(3):357-62. doi: 10.1111/iwj.12243. Epub 2014 Feb 25. PMID: 24612792;PMCID: PMC7950697	Bundles that did not declare the stage of development
Implementing the central venous catheter infection prevention bundle in the emergency department: experiences among early adopters. Ann Emerg Med. 2014 Mar;63(3):340-50.e1. doi: 10.1016/j.annemergmed.2013.09.006. Epub 2013 Oct 10. PMID: 24120627	Bundles that did not declare the stage of development
Prevention of central line associated bloodstream infections in critical care units. Curr Opin Pediatr. 2011 Feb;23(1):85-90. doi: 10.1097/MOP.0b013e328341d1da. PMID: 21124224	Bundles that did not declare the stage of development
Implementation of the ABCDEF Bundle for Critically Ill ICU Patients During the COVID-19 Pandemic: A Multi-National 1-Day Point Prevalence Study	Bundles that did not declare the stage of development
Impact of an evidence-based bundle intervention in the quality-of-care management and outcome of Staphylococcus aureus bacteremia. Clin Infect Dis. 2013 nov;57(9):1225-33. doi: 10.1093/cid/cit499. Epub 2013 Aug 8. PMID: 23929889	Bundles that did not declare the stage of development
Using a multimodal strategy to improve patient hand hygiene	Bundles that did not declare the stage of development
Implementation of an evidence-based bundle to reduce surgical site infection after caesarean section – Review of the interventions	Bundles that did not declare the stage of development
Implementation of an evidence-based bundle to reduce surgical site infection after caesarean section - Review of the interventions. Am J Infect Control. 2022 Oct;50(10):1103-1109. doi: 10.1016/j.ajic.2022.04.005. PMID: 36150794	Bundles that did not declare the stage of development
Meta-analysis of the central line bundle for preventing catheter-related infections: a case study in appraising the evidence in quality improvement. BMJ Qual Saf. 2016 Feb;25(2):118-29. doi: 10.1136/bmjqs-2014-003787. Epub 2015 Jul 16. PMID:26183714	Bundles that did not declare the stage of development
The ABCDEF Bundle in Critical Care. Crit Care Clin. 2017 Apr;33(2):225-243. doi: 10.1016/j.ccc.2016.12.005. PMID: 28284292; PMCID: PMC5351776.	Bundles that did not declare the stage of development
Impact of a program to prevent central line-associated bloodstream infection in the zero tolerance era. Am J Infect Control. 2010 Aug;38(6):434-9. doi: 10.1016/j.ajic.2009.11.012. Epub 2010 Mar 12. PMID:20226570	Bundles that did not declare the stage of development
A Protocol-Driven Reduction in Surgical Site Infections After Colon Surgery	Bundles that did not declare the stage of development
Care bundles: the holy grail of infectious risk management in hospital? Curr Opin Infect Dis. 2009 Aug;22(4):364-9. doi: 10.1097/QCO.0b013e32832e0736. PMID: 19506477	Bundles that did not declare the stage of development
Prevention of healthcare-associated infections in low-and middle-income Countries: The 'bundle approach'	Bundles that did not declare the stage of development

Studies	Reasons for exclusion
Transferring central line care evidence into practice on pediatric acute care units	Bundles that did not declare the stage of development
Methicillin-resistant Staphylococcus aureus prevention practices in hospitals throughout a rural state	Bundles that did not declare the stage of development
Methicillin-resistant Staphylococcus aureus prevention practices in hospitals throughout a rural state. Am J Infect Control. 2014 Aug;42(8):868-73. doi: 10.1016/j.ajic.2014.05.004. PMID: 25087139	Bundles that did not declare the stage of development
Central line insertion bundle: experiences and challenges in an adult ICU. Nurs Crit Care. 2012 May- Jun;17(3):123-9. doi: 10.1111/j.1478-5153.2012.00491.x. Epub 2012 Mar 1. PMID:22497916	Bundles that did not declare the stage of development
Reducing unnecessary urinary catheter use and other strategies to prevent catheter-associated urinary tract infection: an integrative review	Bundles that did not declare the stage of development
Reducing unnecessary urinary catheter use and other strategies to prevent catheter-associated urinary tract infection: an integrative review. BMJ Qual Saf. 2014 Apr;23(4):277-89. doi: 10.1136/bmjqs-2012-001774. Epub 2013 Sep 27. PMID: 24077850; PMCID: PMC3960353	Bundles that did not declare the stage of development
The effect of implementing an aseptic practice bundle for anaesthetists to reduce postoperative infections, the Anaesthetists Be Cleaner (ABC) study: protocol for a stepped wedge, cluster randomised, multi-site trial	Bundles that did not declare the stage of development
Decreasing PICU catheter-associated bloodstream infections: NACHRI's quality transformation efforts...National Association of Children's Hospitals and Related Institutions	Bundles that did not declare the stage of development
Decreasing PICU catheter-associated bloodstream infections: NACHRI's quality transformation efforts. Pediatrics. 2010 Feb;125(2):206-13. doi: 10.1542/peds.2009-1382. Epub2010 Jan 11. PMID: 20064860	Bundles that did not declare the stage of development
Systems initiatives reduce healthcare-associated infections: A study of 22,928 device days in a single trauma unit	Bundles that did not declare the stage of development
Pediatric central line: bundle implementation and outcomes. J Infus Nurs. 2011 Jan-Feb;34(1):36-48. doi: 10.1097/NAN.0b013e31820233d0. PMID: 21239950	Bundles that did not declare the stage of development
Implementing evidence-based nursing practice in the pediatric intensive care unit. J Infus Nurs. 2007 Mar-Apr;30(2):105-12; quiz 120-1. doi: 10.1097/01.NAN.0000264714.20381.d0. PMID: 17413495	Bundles that did not declare the stage of development
Reducing ventilator-associated pneumonia in intensive care: impact of implementing a care bundle	Bundles that did not declare the stage of development
Preventing ventilator-associated events: complying with evidence-based practice. Crit Care Nurs Q. 2014 Oct-Dec;37(4):384-92. doi: 10.1097/CNQ.0000000000000039. PMID: 25185766	Bundles that did not declare the stage of development
Multidisciplinary quality improvement initiative to reduce ventilator-associated tracheobronchitis in the PICU	Bundles that did not declare the stage of development
Multidisciplinary quality improvement initiative to reduce ventilator-associated tracheobronchitis in the PICU. Pediatr Crit Care Med. 2013 Jun;14(5):533-8. doi: 10.1097/PCC.0b013e31828a897f. PMID: 23628838; PMCID: PMC3847907	Bundles that did not declare the stage of development
What is the Role of Nursing Homes in the Surviving Sepsis Campaign?	Bundles that did not declare the stage of development
Use of ventilator bundle to prevent ventilator associated pneumonia	Bundles that did not declare the stage of development
The ABCDE bundle implementation in an intensive care unit: Facilitators and barriers perceived by nurses and doctors	Bundles that did not declare the stage of development
Early progressive mobilization in the intensive care unit with out dedicated personnel	Bundles that did not declare the stage of development
Combination of Oral Antibiotics and Mechanical Bowel Preparation Reduces Surgical Site Infection in Colorectal Surgery. J Am Coll Surg. 2017 Oct;225(4):465-471. doi: 10.1016/j.jamcollsurg.2017.06.011. Epub 2017 Jul 6. PMID: 28690206	Bundles that did not declare the stage of development

Studies	Reasons for exclusion
Preventing Ventilator-Associated Tracheitis in the Pediatric Cardiac Intensive Care Unit.,"Olsen, Jessica	Bundles that did not declare the stage of development
The incidence of ventilator-associated pneumonia (VAP) in a tertiary-care center: Comparison between pre- and post-VAP prevention bundle	Bundles that did not declare the stage of development
The incidence of ventilator-associated pneumonia (VAP) in a tertiary-care center: Comparison between pre- and post-VAP prevention bundle. J Infect Public Health. 2020 Apr;13(4):552-557. doi: 10.1016/j.jiph.2019.09.015. Epub 2019 Oct 25. PMID: 31668986	Bundles that did not declare the stage of development
Ventilator-associated pneumonia and role of nurses in its prevention	Bundles that did not declare the stage of development
A Multifaceted Approach to the Prevention of Clostridioides (Clostridium) Difficile. Clin Nurse Spec. 2019 Mar/Apr;33(2):75-81. doi: 10.1097/NUR.0000000000000426. PMID: 30730451	Bundles that did not declare the stage of development
Evidence-based measures to prevent central line-associated bloodstream infections: a systematic review. Rev Lat Am Enfermagem. 2016 Sep 1;24:e2787. doi: 10.1590/1518-8345.1233.2787. PMID:27598378; PMCID: PMC5016007	Bundles that did not declare the stage of development
Evaluation of a bundle of care to reduce incisional surgical site infection after gastrointestinal surgery	Bundles that did not declare the stage of development
Reduction of central-line-associated bloodstream infection (CLABSI) in resource limited, nonintensive care unit (ICU) settings	Bundles that did not declare the stage of development
A bundle strategy including patient hand hygiene to decrease clostridium difficile infections	Bundles that did not declare the stage of development
A bundle strategy including patient hand hygiene to decrease clostridium difficile infections. Medsurg Nurs. 2014 May-Jun;23(3):145-8, 164. PMID: 25137788	Bundles that did not declare the stage of development
Colorectal bundles for surgical site infection prevention: A systematic review and meta-analysis	Bundles that did not declare the stage of development
Relationship Between a Sepsis Intervention Bundle and In-Hospital Mortality Among Hospitalized Patients: A Retrospective Analysis of Real-World Data. Anesth Analg. 2017 Aug;125(2):507-513. doi: 10.1213/ANE.0000000000002085. PMID:28514322	Bundles that did not declare the stage of development
Effectiveness of insertion and maintenance bundles in preventing peripheral intravenous catheter-related complications and bloodstream infection in hospital patients: A systematic review	Bundles that did not declare the stage of development
Effectiveness of insertion and maintenance bundles in preventing peripheral intravenous catheter-related complications and bloodstream infection in hospital patients: Asystematic review. Infect Dis Health. 2019 Aug;24(3):152-168. doi: 10.1016/j.idh.2019.03.001. Epub 2019 Apr 18. PMID: 31005606	Bundles that did not declare the stage of development
Reducing external ventricular drain associated ventriculitis: An improvement project in a level 1 trauma center	Bundles that did not declare the stage of development
Improving the quality of the intensive care follow-up of ventilated patients during a national registration program. Public Health. 2017 Jul;148:159-166. doi: 10.1016/j.puhe.2017.03.014. Epub 2017 May 11. PMID: 28501761	Bundles that did not declare the stage of development
Using a Bundle Approach to Improve Ventilator Care Processes and Reduce Ventilator- Associated Pneumonia	Bundles that did not declare the stage of development
Beyond the central line-associated bloodstream infection bundle: the value of the clinical nurse specialist in continuing evidence-based practice changes. Clin Nurse Spec. 2012 Jul-Aug;26(4):205-11. doi: 10.1097/NUR.0b013e31825aebab. PMID: 22678186	Bundles that did not declare the stage of development
Trends in ventilator-associated pneumonia: Impact of a ventilator care bundle in an Italian tertiary care hospital intensive care unit	Bundles that did not declare the stage of development
Determining impacts and factors in ventilator-associated pneumonia bundle	Bundles that did not declare the stage of development
External ventriculostomy-associated infection reduction after updating a care bundle	Bundles that did not declare the stage of development

Studies	Reasons for exclusion
International nosocomial Infection Control Consortium (INICC) resources: INICC multidimensional approach and INICC surveillance online system. Am J Infect Control. 2016 Jun 1;44(6):e81-90. doi: 10.1016/j.ajic.2016.01.005. Epub 2016 Mar 11. PMID: 26975716	Bundles that did not declare the stage of development
Effect of the application of a bundle of three measures (intraperitoneal lavage with antibiotic solution, fascial closure with Triclosan-coated sutures and Mupirocin ointment application on the skin staples) on the surgical site infection after elective laparoscopic colorectal cancer surgery	Bundles that did not declare the stage of development
L'utilizzo dei bundle nella pratica clinica [The use of bundles in clinical practice]. Infez Med. 2008 Sep;16(3):121-9. Italian. PMID: 18843209	Bundles that did not declare the stage of development
Best practices for disinfection of noncritical environmental surfaces and equipment in health care facilities: A bundle approach	Bundles that did not declare the stage of development
Best practices for disinfection of noncritical environmental surfaces and equipment in health care facilities: A bundle approach. Am J Infect Control. 2019 Jun;47S:A96-A105. doi:10.1016/j.ajic.2019.01.014. PMID: 31146858.	Bundles that did not declare the stage of development
Sustained Reduction and Prevention of Neonatal and Pediatric Central Line-Associated Bloodstream Infection Following a Nurse-Driven Quality Improvement Initiative in a Pediatric Facility	Bundles that did not declare the stage of development
Central venous catheter care for the patient with cancer: American Society of Clinical Oncology clinical practice guideline	Bundles that did not declare the stage of development
Recognizing and managing severe sepsis: a common and deadly threat. South Med J. 2007 Jun;100(6):594-600. doi: 10.1097/SMJ.0b013e31804aa29f. PMID: 17591313	Bundles that did not declare the stage of development
Development of a statewide collaborative to decrease NICU central line-associated bloodstream infections	Bundles that did not declare the stage of development
Impact of an evidence-based intervention on urinary catheter utilization, associated process indicators, and infectious and non-infectious outcomes. J Hosp Infect. 2020 Oct;106(2):364-371. doi: 10.1016/j.jhin.2020.07.002. Epub 2020 Jul 10. PMID:32653433	Bundles that did not declare the stage of development
Prevention of Device-Related Healthcare-Associated Infections	Bundles that did not declare the stage of development
Kamishibai cards to sustain evidence-based practices to reduce healthcare-associated infections. Am J Infect Control. 2019 Apr;47(4):358-365. doi: 10.1016/j.ajic.2018.10.004. Epub 2018 Dec 4. PMID: 30522838	Bundles that did not declare the stage of development
Central line bundle maintenance among adults in a university hospital intensive care unit in São Paulo, Brazil: a best practice implementation project. JBI Database System Rev Implement Rep. 2018 Jun;16(6):1454-1473. doi: 10.11124/JBISRIR-2017-003561. PMID: 29894411	Bundles that did not declare the stage of development
The IMPROVE-GAP Trial aiming to improve evidence-based management of community-acquired pneumonia: study protocol for a stepped-wedge randomised controlled trial. Trials. 2018 Feb 5;19(1):88. doi:10.1186/s13063-017-2407-4. PMID: 29402313; PMCID: PMC5800278	Bundles that did not declare the stage of development
Are central line bundles and ventilator bundles effective in critically ill neonates and children? Intensive Care Med. 2013 Aug;39(8):1352-8. doi: 10.1007/s00134-013-2927-7. Epub 2013 Apr 25. PMID:23615702	Bundles that did not declare the stage of development
Implementation of a Standardized Patient/Family Communication Bundle	Bundles that did not declare the stage of development
Implementation of a Standardized Patient/Family Communication Bundle. Crit Care Nurs Clin north Am. 2020 Jun;32(2):243-251. doi: 10.1016/j.cnc.2020.02.006. Epub 2020 Mar 29. PMID:32402319	Bundles that did not declare the stage of development
The effect of the ABCDE/ABCDEF bundle on delirium, functional outcomes, and quality of life in critically ill patients: A systematic review and meta-analysis. Int J Nurs Stud. 2023 Feb;138:104410. doi: 10.1016/j.ijnurstu.2022.104410. Epub 2022 nov 29. PMID: 36577261	Bundles that did not declare the stage of development
Compliance with evidence-based guidelines for the prevention of central line-associated bloodstream infections in a Belgian home care setting: An observational study. Am J Infect Control. 2019 Jun;47(6):723-725. doi: 10.1016/j.ajic.2018.10.019. Epub 2018 Dec 6. PMID: 30527768	Bundles that did not declare the stage of development
Characterising an implementation intervention in terms of behaviour change techniques and theory: the 'Sepsis Six' clinical care bundle	Bundles that did not declare the stage of development
The implementation of an external ventricular drain care bundle to reduce infection rates. Br J Neurosurg. 2020 Apr;34(2):181-186. doi:10.1080/02688697.2020.1725436. Epub 2020 Feb 11. PMID: 32046516	Bundles that did not declare the stage of development

Studies	Reasons for exclusion
Do surgical care bundles reduce the risk of surgical site infections in patients undergoing colorectal surgery? A systematic review and cohort meta-analysis of 8,515 patients	Bundles that did not declare the stage of development
Do surgical care bundles reduce the risk of surgical site infections in patients undergoing colorectal surgery? A systematic review and cohort meta-analysis of 8,515 patients. Surgery. 2015 Jul;158(1):66-77. doi: 10.1016/j.surg.2015.03.009. Epub2015 Apr 25. PMID: 25920911	Bundles that did not declare the stage of development
Prevention of central venous catheter-related infection in the neonatal unit: a literature review. J Matern Fetal Neonatal Med. 2015 Jul;28(10):1224-30. doi: 10.3109/14767058.2014.949663. Epub 2014 Sep 26. PMID: 25076387	Bundles that did not declare the stage of development
Increasing Evidence-Based Interventions in Patients with Acute Infections in a Resource-Limited Setting: A Before-and-After Feasibility Trial in Gitwe, Rwanda. Crit Care Med. 2018 Aug;46(8):1357-1366. doi:10.1097/CCM.0000000000003227.PMID: 29957715	Bundles that did not declare the stage of development
New Nutrition Guidelines: Promoting Enteral Nutrition via a Nutrition Bundle	Bundles that did not declare the stage of development
APPLICATION OF A PNEUMONIA PREVENTION BUNDLE IN A PEDIATRIC ICU	Bundles that did not declare the stage of development
Impact of a bundle on surgical site infections after hip arthroplasty: A cohort study in Italy (2012-2019)	Bundles that did not declare the stage of development
Paramedic-Initiated CMS Sepsis Core Measure Bundle Prior to Hospital Arrival: A Stepwise Approach. Prehosp Emerg Care. 2017 May-Jun;21(3):291-300. doi: 10.1080/10903127.2016.1254694. Epub 2016 Dec 5. PMID: 27918869	Bundles that did not declare the stage of development
Reduction of central line-associated bloodstream infection rates in patients in the adult intensive care unit	Bundles that did not declare the stage of development
Prevention of peripherally inserted central line-associated blood stream infections in very low-birth-weight infants by using a central line bundle guideline with a standard checklist: a case control study. BMC Pediatr. 2015 Jun 18;15:69. doi: 10.1186/s12887-015-0383-y. PMID: 26084807; PMCID: PMC4470111	Bundles that did not declare the stage of development
Practical Anemia Bundle for Sustained Blood Recovery (PABST-BR) in critical illness: A protocol for a randomised controlled trial	Bundles that did not declare the stage of development
Effectiveness of a multidisciplinary patient care bundle for reducing surgical-site infections	Bundles that did not declare the stage of development
Eliminating catheter-associated urinary tract infections: part I. Avoid catheter use	Bundles that did not declare the stage of development
Eliminating catheter-associated urinary tract infections: part I. Avoid catheter use. J Healthc Qual. 2009 nov-Dec;31(6):8-12. doi: 10.1111/j.1945-1474.2009.00049.x.PMID: 19957458	Bundles that did not declare the stage of development
Implementation and evaluation of a care bundle for prevention of non-ventilator-associated hospital-acquired pneumonia (nvHAP) - a mixed-methods study protocol for a hybrid type 2 effectiveness-implementation trial	Bundles that did not declare the stage of development
Evidence-based Care Bundles for Preventing Surgical Site Infections in Spinal Instrumentation Surgery. Spine (Phila Pa 1976). 2018 Dec 15;43(24):1765-1773. doi: 10.1097/BRS.0000000000002709. PMID: 29794586	Bundles that did not declare the stage of development
Efficacy of a care bundle to prevent multiple infections in the intensive care unit: A quasi-experimental pretest-posttest design study. Appl Nurs Res. 2018 Feb;39:4-10. doi: 10.1016/j.apnr.2017.10.009. Epub 2017 Oct 17. PMID: 29422174	Bundles that did not declare the stage of development
Comparison of three interventional approaches to prevent ventilator-associated pneumonia in intensive care units (ICUs): A clinical trial study	Bundles that did not declare the stage of development
Evidence-based interventions to reduce adverse events in hospitals: a systematic review of systematic reviews	Bundles that did not declare the stage of development
Bundles Prevent Surgical Site Infections After Colorectal Surgery: Meta-analysis and Systematic Review. J Gastrointest Surg. 2017 nov;21(11):1915-1930. doi: 10.1007/s11605-017-3465-3. Epub 2017 Jun 15. PMID: 28620749	Bundles that did not declare the stage of development
CONSTRUÇÃO DE UM BUNDLE PARA PREVENÇÃO DE INFECÇÃO DE CORRENTE SANGUÍNEA ASSOCIADA AO CATETER VENOSO CENTRAL	Bundles that did not declare elements

Studies	Reasons for exclusion
Using care bundles to prevent infection in neonatal and paediatric ICUs. Curr Opin Infect Dis. 2009 Jun;22(3):224-8. doi:10.1097/QCO.0b013e3283297b68. PMID: 19369867	Bundles that did not declare elements
Reducing the incidence of stroke-associated pneumonia: an evidence-based practice	Bundles that did not declare elements
Prevention of Clostridium difficile Infection: A Systematic Survey of Clinical Practice Guidelines	Bundles that did not declare elements
Surviving Sepsis Campaign: International Guidelines for Management of Sepsis and Septic Shock: 2016. Intensive Care Med. 2017 Mar;43(3):304-377. doi:10.1007/s00134-017-4683-6. Epub 2017 Jan 18. PMID: 28101605	Bundles that did not declare elements
Colorectal Delphi Facilitating Group. Delphi Consensus on Intraoperative Technical/Surgical Aspects to Prevent Surgical Site Infection after Colorectal Surgery. J Am Coll Surg. 2022 Jan 1;234(1):1-11. doi: 10.1097/XCS.0000000000000022.PMID: 35213454; PMCID: PMC8719508	Bundles that did not declare elements
