# Supplementary material for: Identifying the Bundle/Care Development Process in Clinical Risk Management: A Systematic Review
Source: Healthcare (Basel). 2024 Nov 11;12(22):2242. doi: 10.3390/healthcare12222242 (PMC11593500; doi:10.3390/healthcare12222242)
Supplement: Supplementary file 1 [file healthcare-12-02242-s001.zip › Multimedia File S3 (Summary of finding of included studies).pdf]

Multimedia File S3 (Summary of findings of included studies)

Summary of findings of included studies

N.	Study	Author	Date	Country	DB	Study design	Situation risk	Methods of developing	Components of the bundle	Qualitative Valutation
1	Assessing the Efficacy of Ventilator-Associated Event Prevention Bundle in the Intensive Care Units: An Intervention Study	Acun A.	2022	Turkey	Cinhal	Intervention Study	BUNDLE VAP	1. CDC criteria and guidelines,<br>2. Guidelines prepared by the General Directorate of Public Health affiliated to Republic of Turkey Ministry of Health<br>3. Literature review<br>4. Opinions of one infection control doctor and four infection control nurses, who were experts in their fields	1. Head of the bed elevation should be at an angle of 30-45 degrees<br>2. Use of an oral chlorhexidine rinse<br>3. Aseptic aspiration should be applied<br>4. The ventilator circuits are not dirty	Inclusion
2	Decreased Surgical Site Infection Rate in Hysterectomy: Effect of a Gynecology-Specific Bundle	Andiman S.E.	2018	United States of America	Cinhal	Quality improvement study	BUNDLE SSI	1. The bundle was designed and implemented by the committee led by representatives from gynecology, anesthesia, hospital epidemiology and infection control, and peri- and postoperative nursing leadership.<br>2. The group developed a gynecology-specific bundle based on existing evidence and best practice guidelines, some of which were extrapolated from colorectal and other surgery literature	1. Chlorhexidine gluconate<br>2. Patient-controlled preoperative warming<br>3. Aseptic gynecologic skin preparation<br>4. Sterile dressing<br>5. Maintenance of intraoperative warming<br>6. Antibiotic standardization and redosing<br>7. Direct feedback regarding bundle compliance	Inclusion
3	Preventing catheter-associated urinary tract infections in acute care: the bundle approach	Andreessen L.	2012	United States of America	Cinhal	Quality improvement study	BUNDLE CAUTI	1.CDC criteria and guidelines<br>2. A multidisciplinary team was assembled. The team included 2 infection control nurses, 2 nurse educators, 3 staff nurses, 2 surgical nurses, 4 nurse managers (operating room, ER, ICU,and medical units), 2 clinical nurse leaders, 1 urologist, 2 information technologists, and 1 chief medical resident.	Insertion<br>1. Obtain provider order for insertion. Indications included in orders template, based on 2009 CDC guidelines.<br>2. Obtain urinary catheter and/or appropriate closed drainage system. Smaller size urinary catheter preferred (eg, 12-14); larger sizes inserted by urologist.<br>3. Strict hand hygiene. Strict aseptic technique performed for every insertion with 2 staff present.<br>4. Secure catheter to patient thigh or abdomen using securing device.<br>5. Document insertion date and urinary catheter size.<br>Maintenance<br>1. Assess daily need for ongoing catheter.<br>2. Consider alternatives to indwelling urethral catheters (eg, condom catheter, straight	Inclusion

									catheterization, use of bladder scanner, or bladder training program).<br>3. For surgical patients with catheter >48 h, providers must document daily clinical need.<br>4. Perform pericare daily and after each bowel movement.<br>5. Keep drainage bag and tubing below level of bladder and off floor.<br>6. Confirm presence and attachment of catheter securing device every shift.<br>7. Ensure no kinks in drainage tubing and do not clamp >2 hours for specimen collection.<br>8. When sending C&S sample, cleanse sampling port vigorously with alcohol and allow to air dry;<br>label sample as catheter obtained.	
4	Evaluating an evidence-based bundle for preventing surgical site infection: a randomized trial.	Anthony T.	2011	United States of America	PubMed	Randomized controlled trial	BUNDLE SSI	1. A review of the literature was conducted that focused on evidence-based measures that could be expected to reduce SSI and could be instituted in our practice setting.	1. omission of mechanical bowel preparation8<br>2. use of preoperative and intraoperative warming designed to maintain normothermia<br>3. maintenance of increased concentration of inspired oxygen during and immediately after surgery<br>4.reduction of intravenous fluids during the operation;<br>5. the use of wound edge protection"	Esclusion
5	An audit of compliance with the sepsis resuscitation care bundle in patients admitted to A&E with severe sepsis or septic shock.	Baldwin L.N.	2008	United Kingdom	PubMed	Observational study	BUNDLE SEPSIS	1. Surviving Sepsis campaign guidelines for the management of severe sepsis and septic shock<br>2. Development of the package with consensus (Delphi consensus method)	1. Fluid challenge (20 ml/kg crystalloid immediately) with subsequent fluid therapy according to the protocol of Rivers et al. (2001).<br>2. Blood to be taken for culture before antibiotic administration.<br>3. First dose of an appropriate antibiotic to be given within 3 h of admission to A&E (1 h after onset of sepsis in ward setting).<br>4. Measurement of arterial blood lactate concentration.<br>5. Placement of a central venous line in presence of shock persisting after fluid challenge.<br>6. Measurement of central venous pressure and oxygen saturation. These variables are then targeted to end points using fluid and pressor agents.<br>All elements to be completed along with predetermined haemodynamic targets within 6 h of admission to A&E (or onset of sepsis in a ward setting).	Esclusion

6	A flowchart for building evidence-based care bundles in intensive care: based on a systematic review.	Borgert M.	2017	Netherlands	PubMed	Systematic review	BUNDLE VAP; BUNDLE CLABSI	1. Identify problems or risks in a specific patient population or intervention that contributes to great harm and/or high cost (Systematic reviews- Adverse Event Trigger Tool)<br>2. The identified care problems or risks should be clearly defined (Comprehensive literature search strategy)<br>3. Conduct a literature search to collect relevant evidence for the problems or risks and to find related elements (Collect evidence from the international electronic databases and from the distillation from inter-national clinical guidelines)<br>4. Select potential relevant and feasible elements from the literature search (Select those elements that were described in the literature and were associated with the identified problem or from local or (inter) national clinical guidelines)<br>5.Select a final set of maximally five elements (GRADE approach to evaluate the quality of the evidence of the elements) (Weighing and scoring technique to select the most suitable, reliable or most appropriate key elements root cause analyses; FMEA; through discussion sessions or consensus meetings with experts or hospital staff)<br>6.Create the care bundle in draft form (Create the bundle in draft form and check if the IHI bundle requirements are met)<br>7. Pilot test the care bundle in order to assess the reliability (The pilot should be performed in a small sample of patients to identify (potential) risks or barriers for implementation. It is important to monitor the	<u>Care Bundle Clabsi</u><br>1.Hand hygiene;<br>2. Maximal barrier precautions;<br>3. Chlorhexidine skin antisepsis;<br>4. Optimal catheter site selection, with avoidance of using the femoral vein for central venous access in adult patients; and<br>5. Daily review of line necessity, with prompt removal of unnecessary lines.<br><u>Care Bundle VAP</u><br>1.Elevation of the head of the bed (HOB) to between 30 and 45 degrees<br>2. Daily —sedative interruption‖ and daily assessment of readiness to extubate<br>3. Peptic ulcer disease (PUD) prophylaxis<br>4. Deep venous thrombosis (DVT) prophylaxis (unless contraindicated)	Inclusion

								performance of all bundle elements to identify potential problems or risks and to evaluate if the care bundle is feasible, comprehensive, effective and easy to use)		
7	Impact of nurse-initiated ed sepsis protocol on. Compliance with sepsis bundles, time to initial antibiotic administration, and in-hospital mortality	Bruce H.R.	2015	United States of America	Cinhal	Observational study (retrospective cohort)	BUNDLE SEPSIS	1. SSC guidelines<br>2. A multidisciplinary health care team to implement the 2008 and 2012 SSC guidelines<br>3. Adaptation of the hospital protocol	1. Measure the serum lactate level<br>2. Obtain blood cultures before antibiotic initiation,<br>3. Administer broad- spectrum antibiotics<br>4. Infuse 30 mL/kg of an intravenous (IV) crystalloid solution in patients with hypotension or a lactate level of 4 mmol/L or greater<br>The Bundle be completed within 3 hours of the ED admission of a patient with sepsis	Inclusion
8	A Bundle Protocol to Reduce the Incidence of Periprosthetic Joint Infections After Total Joint Arthroplasty: A Single-Center Experience	Bullock M.W.	2017	United States of America	Cinhal	Quality improvement study	BUNDLE INFECTIONS	1. In 2011, our institution conducted a comprehensive review of primary TJA cases<br>2. Creation of a multidisciplinary team focused on formulating a "bundle" to optimize patient outcomes. Our team included 3 fellowship-trained TJA surgeons, anesthesiologists, infectious disease specialists, nurses, physical therapists, and administrative coordinators.	Preoperative<br>1.Body mass index <40 kg/m2 Hemoglobin A1c<7.0%<br>2.Tobacco smoking<0.5 packs/day<br>3.Chlorhexidine wash<br>4.Instruction MRSA screen<br>5.Risk factor labs<br>6.Preanesthesia appointment<br>7.Surgeon risk factor assessment<br>Intraoperative<br>1.Hair clipping in holding room<br>2.Chlorhexidine wash<br>3.Isopropyl alcohol wash Minimize OR traffic<br>4.Exchange gloves before implanting<br>5.No "flashed" instruments<br>6.Dilute povidone-iodine solution wash<br>7.Silver-impregnated dressing<br>Postoperative<br>1. Dedicated total joint unit<br>2. 24 h of antibiotics<br>3.Standard wound care Chlorhexidine wash<br>4. Aspirin for low-risk patients<br>5. Follow-up instruction phone call	Inclusion

9	Reducing abdominal hysterectomy surgical site infections: A multidisciplinary quality initiative	Davidson C.	2020	United States of America	Cinhal	Retrospective cohort study	BUNDLE SSI	1.CDC criteria and guidelines	Preoperative<br>1. At the pre-op clinic visit, patients should be informed of their specific risk factors for development of SSI and risk reduction strategies.<br>2.At the pre-op Anesthesia/Nursing visit<br>Pre-op holding/PACU<br>1. Normothermic control: forced warm air gown to maintain normothermia for patients prior to surgery (36-37C)<br>2. CHG wipes if no CHG shower morning of surgery<br>3. Check blood glucose level<br>Intraoperative<br>1. Surgeon hand/forearm antisepsis: follow guidelines for specific product chosen<br>2. Administer prophylactic antibiotic within one hour prior to surgical incision and re-dose based on blood loss and length of operation<br>3. Prep skin with CHG and prep vagina with PI<br>4. Remove hair immediately before operation with electric clippers rather than shaving, if hair removal is indicated<br>5. Surgical/operating room attire<br>6. Ensure double-gloving for all scrubbed surgical team members<br>7. Maintain perioperative normothermia<br>8. Anesthesia to check blood glucose at anesthesia induction and again 1 hour into hysterectomy procedures and administer insulin for any blood glucose level >140 mg/dL<br>9. Closing protocol at time of fascia closure<br>10. Ensureallnon-sterileindividualsremain12-18inches away from the sterile field<br>11. Limit traffic of all persons entering or exiting the operating room<br>Postoperative<br>1. Maintain control of serum blood glucose levels in all diabetic patients<br>2. Post-op wound care<br>3. Discharge education<br>4. Complete checklist for each patient to monitor compliance with bundle components	Inclusion

10	Impact of a surgical site infection bundle on cesarean delivery infection rates.	Davidson C.	2020	United States of America	PubMed	Retrospective cohort study	BUNDLE SSI	1.Using CDC guidelines<br>2. Integration into the CDC guidelines of the results of a review of the literature on colorectal surgery in which the reduction in superficial SSI rates was demonstrated	Preoperative<br>1.Chlorhexidine bathing<br>2. Blood glucose analysis/treatment in diabetics<br>Intraoperative<br>1.Appropriate antibiotic usage<br>2. Blood glucose analysis/treatment in diabetics<br>3. Appropriate surgical site preparation<br>4. Double-gloving by all scrubbed staff<br>5. Fascial closure procedure<br>Postoperative<br>1. Dressing removal on 2nd postoperative day<br>2. Blood glucose analysis/treatment in diabetics<br>3. Discharge education and instructions given<br>4. Discharge phone calls	Inclusion

11	Implementation of a comprehensive unit-based safety program to reduce surgical site infections in cesarean delivery	Dieplinger B.	2020	Austria	Cinhal	Observational study	BUNDLE SSI	1.Initiation of a Comprehensive Unit-based Safety Program (CUSP)<br>2.We introduced a bundle of evidence-based interventions<br>3.We implemented the evidence-based bundle using the CUSP approach into clinical routine	Strict compliance with hygiene standards and practice good hand hygiene throughout the whole perioperative process<br>Preoperative process<br>Outpatient appointment<br>1.The women were instructed about not shaving the intimate area three days before the planned cesarean delivery<br>2.Pathologic glucose tolerance and/or gestational diabetes were recorded and treated<br>3.The women were instructed to take a shower in the morning of the day of planned cesarean delivery<br>Delivery room<br>1.Hair removal with clippers at the incision site if necessary<br>2.Maintaining normothermia (>36°C)<br>3.Glycemic control (<200 mg/dl)<br>4.Weight adjusted antibiotic prophylaxis 30 min before skin incision<br><120 kg body weight cephalosporin 2 g<br>>120 kg body weight cephalosporin 3 g<br>Intraoperative process<br>Surgery room<br>1.Checking if weight-adjusted antibiotic prophylaxis was given correctly (repeated redose after 1,500 ml of blood loss and/or at 3 hours of operating time)<br>2.Maintaining normothermia (>36°C)<br>3.Glycemic control (<200 mg/dl)<br>4.Suture closure of subcutaneous tissue thickness ≥2 cm<br>Postoperative process<br>Surgery recovery room<br>1.Maintaining normothermia (>36°C)<br>2.Glycemic control (<200 mg/dl)<br>Hospital ward<br>3.Indwelling urinary catheter removal within 24 hours after cesarean delivery<br>4.No routine change of the surgical wound dressing within 48 hours after the cesarean delivery<br>5.Handouts and instructions for peripartum behavior for women before discharge	Inclusion

12	Does our bundle stack up! Innovative nurse-led changes for preventing catheter-associated urinary tract infection (CAUTI)	Giles M.	2015	Australia	Cinhal	Interventional study	BUNDLE CAUTI	1.Exploration of the literature 2.Collaboration with all stakeholders and development of evidence-based IUC insertion criteria, care bundles and guidelines for the nurse-led protocols. 3.Further consultation with ward staff related to implementation strategies, nomination of ward champions to engage ward staff and assist in implementation of the nurse-led protocol	1.NEED- for catheter assessed- refer to indications,scan bladder, considser alternative, document reason 2.OBTAIN- patient consent, OFFER patient education 3.COMPETENCY-clinicians who insert catheters must have documented competency 4.ASEPSIS- maintain asepsis during insertion and while catheter is in place 5.UNOBSTRUCTED flow- No kinks or loops, catheter secured, bag below bladder level and off the floor 6.TIMELY catheter removal and documentation- Nurse initiated (refer to guidelines) 7.INFECTION RISK- collect urine specimen only when clinically indicated	Inclusion
13	Neonatal Intensive Care Unit (NICU) White Paper Series: Practical approaches for the prevention of central-line-associated bloodstream infections.	Muller M.	2023	United States of America	PubMed	White Paper Series (Expert opinion)	BUNDLE CLABSI	1. Practical approaches in question-answer format, with responses based on the consensus opinion of pediatric experts, including pediatric infectious disease specialists, neonatologists, advanced practice nurse practitioners, and infection experts, convened by SHEA using the guidelines as documents HICPAC guidance and other published documents collected from a literature review	Insertion 1.Perform hand hygiene before insertion. 2. Adhere to aseptic technique. 3. Use maximal sterile barrier precautions (i.e., mask, cap, gown, sterile gloves, and sterile full body drape). 4. Choose the best insertion site to minimize infections and noninfectious complications based on individual patient characteristics. 5. Prepare the insertion site with >0.5% CHG with alcohol* (see Question/ Answer 1). 6. Place a sterile gauze dressing or a sterile, transparent, semipermeable dressing over the insertion. 7. For patients ≥18 years of age, use a CHG-impregnated dressing with an FDA cleared label that specifies a clinical indication for reducing CLABSI for short-term non-tunneled catheters unless the facility is demonstrating success at preventing CLABSI with baseline prevention practices* Maintenance 1. Perform hand hygiene. 2. Bathe ICU patients who are ≥2 months of age with CHG daily. 3. Use only sterile devices to access catheters. 4. Scrub the access port or hub with friction immediately prior to each use with an appropriate	Inclusion

									antiseptic (CHG, povidone iodine, an iodophor, or 70% alcohol).<br>5. Immediately replace dressings that are wet, soiled, or dislodged.<br>6. Perform routine dressing changes using aseptic technique with clean or sterile gloves:<br>7. Change gauze dressings at least every 2 days.<br>8. Change semipermeable dressings at least every 7 days.<br>9. For patients >18 years of age, use a chlorhexidine impregnated dressing with an FDA cleared label that specifies a clinical indication for reducing CLABSI for short-term non-tunneled catheters unless the facility is demonstrating success at preventing CLABSI*.<br>10. Change administrations sets for continuous infusions no more frequently<br>than every 4 days, but at least every 7 days.<br>11. If blood or blood products or fat emulsions are administered, change tubing every 24 hours.<br>12. If propofol is administered, change tubing every 6-12 hours or when the vial is changed.<br>13. Perform daily audits to assess if central line is still needed	
14	Using Care Bundles to Improve Health Care Quality	Resar R.	2012	United States of America	IHI	White Paper (Narrative)	BUNDLE VAP; BUNDLE CLABSI	1.Team members from the ICUs participating in the collaborative community<br>2. Definition of the Bundle phases	IHI Ventilator Bundle<br>1. Elevation of the head of the bed to between 30 and 45 degrees<br>2. Daily "sedation vacations" and assessment of readiness to extubate<br>3. Peptic ulcer disease (PUD) prophylaxis<br>4. Deep venous thrombosis (DVT) prophylaxis<br>(Note: A fifth bundle element, "Daily oral care with chlorhexidine," was added in 2010.)<br>IHI Central Line Bundle<br>1.Hand hygiene<br>2.Maximal barrier precautions<br>3.Chlorhexidine skin antisepsis<br>4.Optimal catheter site selection, with avoidance of using the femoral vein for central venous access in adult patients<br>5.Daily review of line necessity, with prompt removal of unnecessary lines	Inclusion

15	Co-development of a transitions in care bundle for patient transitions from the intensive care unit: a mixed-methods analysis of a stakeholder consensus meeting.	Rosgen B.K.	2022	Canada	PubMe d	Mixed-methods study	BUNDLE TRANSITIONS	1.A stakeholder consensus meeting	"1.user centered critical care discharge information pack 2.transfer preparation letter preparing parents for their child's transfer from the picu 3. potentially better practices 4. information blooklet 5. pediatric acut burn discharge planning index"	Inclusion
16	A systematic approach for developing a ventilator-associated pneumonia prevention bundle	Speck K.	2016	United States of America	Cinhal	Literature review Delphi method (Expert opinion)	BUNDLE VAP	1. Identification of potential interventions to include through a review of current guidelines and literature. 2.Implemented a 2-step modified Delphi method to obtain consensus on the final list of interventions. 3. An interdisciplinary group of clinical experts participated in the Delphi process, led by a group of technical experts. 4.Identified 65 possible interventions. Through the Delphi method, the list was narrowed down to 19 interventions that included 5 process measures and 14 structural measures.	Process measures 1. Head of bed elevation: use of a semirecumbent position (≥30°) 2. SATs and SBTs: make a daily assessment of readiness to wean with the use of the SAT and SBT. 3. Oral care: at least 6 times per day. 4. Oral care with chlorhexidine: 2 times per day. 5. Subglottic suctioning: use subglottic suctioning in patients expected to be MV for >72 h Structural measures 1. Use a closed endotracheal tube suctioning system. 2. Change close suctioning catheters only as needed. 3. Change ventilator circuits only if damaged or soiled. 4. Change heat and moisture exchanger every 5-7 d and as clinically indicated. 5. Provide easy access to noninvasive ventilation equipment and institute protocols to promote use. 6. Periodically remove the condensate from circuits, keeping the circuit closed during the removal, taking precautions no to allow condensate to drain toward patient. 7. Use an early mobility protocol. 8. Perform hand hygiene. 9. Avoid supine position. 10. Use standard precautions while suctioning respiratory tract secretions. 11. Use orotracheal intubation instead of nasotracheal. 12. Avoid use of prophylactic systemic antimicrobials. 13. Avoid nonessential tracheal suctioning. 14. Avoid gastric overdistention.	Inclusion
