# Supplementary material for: Identifying the Bundle/Care Development Process in Clinical Risk Management: A Systematic Review
Source: Healthcare (Basel). 2024 Nov 11;12(22):2242. doi: 10.3390/healthcare12222242 (PMC11593500; doi:10.3390/healthcare12222242)
Supplement: Supplementary file 1 [file healthcare-12-02242-s001.zip › Multimedia File S4 (Critical Appraisal Tool).pdf]

Multimedia File S4 (Critical Appraisal Tool)

È stata eseguita una valutazione con il JBI's critical appraisal tools per i 16 articoli inclusi nella revisione. Lo scopo è stato quello di valutare la qualità metodologica dello studio e di determinare in che misura lo studio ha affrontato la possibilità di distorsioni nella sua progettazione, conduzione e analisi. Tutti gli articoli che soddisfano i criteri di inclusione sono stati sottoposti a una valutazione rigorosa da parte di due valutatori critici. I risultati di questa valutazione possono essere utilizzati per l'interpretazione dei risultati dello studio.

Per quanto riguarda l'articolo di Acun et al [22] dalla valutazione è emerso che risulta chiara la "causa" e l'"effetto"; ci sono state più misurazioni dell'esito sia prima che dopo l'intervento/esposizione; il follow-up è stato completo; i risultati sono stati misurati in modo affidabile ed è stata utilizzata un'analisi statistica appropriata. Mentre non risulta presente e specificato nell'articolo se i partecipanti sono stati inseriti in confronti simili o in confronti che hanno ricevuto un trattamento/cura simile, diverso dall'esposizione o dall'intervento di interesse. Inoltre, non era presente un gruppo di controllo.
Per quanto riguarda l'articolo di Andiman et al [12] dalla valutazione è emerso che risulta chiara la "causa" e l'"effetto"; ci sono state più misurazioni dell'esito sia prima che dopo l'intervento/esposizione; i risultati dei partecipanti sono stati inclusi in qualsiasi confronto misurato allo stesso modo; i risultati sono stati misurati in modo affidabile ed è stata utilizzata un'analisi statistica appropriata. Mentre non risulta presente e specificato nell'articolo se i partecipanti sono stati inseriti in confronti simili o in confronti che hanno ricevuto un trattamento/cura simile, diverso dall'esposizione o dall'intervento di interesse. Inoltre, non era presente un gruppo di controllo e il follow non è stato completo
Per quanto riguarda l'articolo di Andreessen et al [13] dalla valutazione è emerso che risulta chiara la "causa" e l'"effetto"; era presente un gruppo di controllo; ci sono state più misurazioni dell'esito sia prima che dopo l'intervento/esposizione; i risultati dei partecipanti sono stati inclusi in qualsiasi confronto misurato allo stesso modo; i risultati sono stati misurati in modo affidabile ed è stata utilizzata un'analisi statistica appropriata. Mentre non risulta presente e specificato nell'articolo se i partecipanti sono stati inseriti in confronti simili o in confronti che hanno ricevuto un trattamento/cura simile, diverso dall'esposizione o dall'intervento di interesse. Inoltre, era ambiguo il follow up e se i risultati dei partecipanti sono stati inclusi in qualsiasi confronto misurato allo stesso modo.
Per quanto riguarda l'articolo di Anthony et al [14] quasi tutti i campi valutati della lista di controllo erano presenti: la randomizzazione è stata utilizzata per l'assegnazione dei partecipanti ai gruppi di trattamento; l'assegnazione ai gruppi di trattamento è stata nascosta; i gruppi di trattamento erano simili senza differenze; i partecipanti erano ciechi all'assegnazione del trattamento; i gruppi di trattamento sono stati trattati in modo identico a parte l'intervento di interesse; il follow-up è stato completo e, in caso contrario, le differenze tra i gruppi in termini di follow-up sono state adeguatamente descritte e analizzate; i partecipanti sono stati analizzati nei gruppi a cui sono stati randomizzati; i risultati sono stati misurati allo stesso modo per i gruppi di trattamento; i risultati sono stati misurati in modo affidabile; è stata utilizzata un'analisi statistica appropriata; il disegno dello studio è stato appropriato e le eventuali deviazioni dal disegno RCT standard (randomizzazione individuale, gruppi paralleli) sono state prese in considerazione nella conduzione e nell'analisi dello studio. I campi ambigui sono: coloro che erogavano il trattamento erano ciechi all'assegnazione del trattamento ed i valutatori degli esiti erano ciechi all'assegnazione del trattamento.
Dalla valutazione dell'articolo di Baldwin et al [23] è emerso che: le caratteristiche demografiche del paziente sono state descritte chiaramente; l'anamnesi del paziente è stata chiaramente descritta e presentata come una linea temporale; le attuali condizioni cliniche del paziente alla presentazione sono state descritte chiaramente; l'intervento o le procedure di trattamento sono stati descritti in modo chiaro; sono stati identificati e descritti eventi avversi (danni) o eventi imprevisti; il caso clinico fornisce lezioni da asporto. Per quanto riguarda i campi ambigui non sono stati descritti chiaramente i test diagnostici o metodi di valutazione e i risultati; anche la condizione clinica post-intervento non è stata descritta chiaramente.
Dalla valutazione dell'articolo di Borgert et al [24] è emerso che: la domanda di revisione è espressa in modo chiaro ed esplicito; i criteri di inclusione erano appropriati per la domanda di revisione; la strategia di ricerca era appropriata; le fonti e le risorse utilizzate per la ricerca degli studi sono state adeguate; i criteri di valutazione degli studi erano appropriati; le raccomandazioni per le politiche e/o le pratiche sono state supportate dai dati riportati; le direttive specifiche per le nuove ricerche erano appropriate. Risultano ambigui i seguenti campi: la valutazione critica è stata condotta da due o più revisori in modo indipendente; esistevano metodi per ridurre al minimo gli errori nell'estrazione dei dati; i metodi utilizzati per combinare gli studi erano appropriati ed è stata valutata la probabilità di bias di pubblicazione.
Dalla valutazione dell'articolo di Bruce et al [15] è emerso che pochi campi risultano chiari: i due gruppi erano simili e reclutati dalla stessa popolazione; le esposizioni sono state misurate in modo simile per assegnare le persone sia ai gruppi esposti che a quelli non esposti, i risultati sono stati misurati in modo valido ed è stata utilizzata un'analisi statistica appropriata. Non risulta essere stata misurata l'esposizione in modo valido e affidabile; non sono stati identificati i fattori confondenti; non sono state indicate strategie per affrontare i fattori confondenti; non sono state utilizzate strategie per affrontare il follow-up incompleto. Risultano ambigui: i gruppi/partecipanti erano privi dell'esito all'inizio dello studio; il follow-up. Per i seguenti motivi l'articolo è stato escluso.
Dalla valutazione dell'articolo Bullock et al [16] emerso che pochi campi risultano chiari: i due gruppi erano simili e reclutati dalla stessa popolazione; le esposizioni sono state misurate in modo simile per assegnare le persone sia ai gruppi esposti che a quelli non esposti; i risultati sono stati misurati in modo valido e affidabile ed è stata utilizzata un'analisi statistica appropriata. Non risulta essere stata misurata: l'esposizione; i fattori confondenti e le strategie per i fattori confondenti e per il follow-up. Risultano ambigui: i gruppi/partecipanti erano privi dell'esito all'inizio dello studio; il tempo di follow-up e le ragioni della perdita di quest'ultimo. Per i seguenti motivi l'articolo è stato escluso
Dalla valutazione dell'articolo di Davidson et al [17] è emerso che: i due gruppi erano simili e reclutati dalla stessa popolazione; le esposizioni sono state misurate in modo simile per assegnare le persone sia ai gruppi esposti che a quelli non esposti; l'esposizione è stata misurata in modo valido e affidabile; i gruppi/partecipanti erano privi dell'esito all'inizio dello studio; i risultati sono stati misurati in modo valido e affidabile ed è stata utilizzata un'analisi statistica appropriata. Risultano ambigui i seguenti campi: sono stati identificati fattori confondenti; sono state indicate strategie per affrontare i fattori confondenti; il tempo di follow-up è stato sufficiente e completo e non sono state descritte ed esplorate le ragioni della perdita di quest'ultimo; Sono state utilizzate strategie per affrontare il follow-up incompleto.

Dalla valutazione dell’articolo di Davidson et al [18] è emerso che: i due gruppi erano simili e reclutati dalla stessa popolazione; le esposizioni sono state misurate in modo simile per assegnare le persone sia ai gruppi esposti che a quelli non esposti; l'esposizione è stata misurata in modo valido e affidabile; sono stati identificati fattori confondenti; i gruppi/partecipanti erano privi dell'esito all'inizio dello studio; i risultati sono stati misurati in modo valido e affidabile; il tempo di follow-up è stato riportato e sufficiente per essere abbastanza lungo da consentire il verificarsi dei risultati; il follow-up è stato completo e, in caso contrario, sono state descritte ed esplorate le ragioni della perdita di follow-up ed è stata utilizzata un'analisi statistica appropriata. Risultano ambigui i seguenti campi: sono state indicate strategie per affrontare i fattori confondenti; sono state utilizzate strategie per affrontare il follow-up incompleto.
Dalla valutazione dell’articolo di Dieplinger et al [25] è emerso che: i due gruppi erano simili e reclutati dalla stessa popolazione; le esposizioni sono state misurate in modo simile per assegnare le persone sia ai gruppi esposti che a quelli non esposti; l'esposizione è stata misurata in modo valido e affidabile; i gruppi/partecipanti erano privi dell'esito all'inizio dello studio; i risultati sono stati misurati in modo valido e affidabile; il tempo di follow-up è stato riportato e sufficiente per essere abbastanza lungo da consentire il verificarsi dei risultati; il follow-up è stato completo e, in caso contrario, sono state descritte ed esplorate le ragioni della perdita di follow-up ed è stata utilizzata un'analisi statistica appropriata. Risultano ambigui i seguenti campi: sono stati identificati fattori confondenti; sono state indicate strategie per affrontare i fattori confondenti; sono state utilizzate strategie per affrontare il follow-up incompleto.
Dalla valutazione dell’articolo di Giles et al [26] è emerso che: risulta chiaro qual è la "causa" e qual è l'"effetto"; I partecipanti sono stati inclusi in confronti simili; ci sono state più misurazioni dell'esito sia prima che dopo l'intervento/esposizione; il follow-up è stato completo e, in caso contrario, le differenze tra i gruppi in termini di follow-up sono state adeguatamente descritte e analizzate; i risultati sono stati misurati in modo affidabile ed è stata utilizzata un'analisi statistica appropriata. Nell’articolo non è presente gruppo di controllo. Risultano ambigui i seguenti campi: i partecipanti sono stati inclusi in confronti che hanno ricevuto un trattamento/cura simile, diverso dall'esposizione o dall'intervento di interesse ed i risultati dei partecipanti sono stati inclusi in qualsiasi confronto misurato allo stesso modo.
Dalla valutazione dell’articolo di Muller et al [19] risultano chiaramente specificati gli autori; gli autori hanno esperienza e conoscenza in quel campo di competenza; gli interessi della popolazione coinvolta sono al centro del parere dell’esperto; il parere dimostra un'argomentazione logicamente difesa a sostegno delle conclusioni tratte e c'è un riferimento alla letteratura esistente. Risulta ambigua l’incongruenza con la letteratura/le fonti logicamente difese.
Dalla valutazione dell’articolo di Resar et al [1] risulta chiaro che: il generatore della narrazione è una fonte credibile o appropriata; viene spiegata la relazione tra il testo e il suo contesto; la narrazione presenta gli eventi utilizzando una sequenza logica in modo che il lettore o l'ascoltatore possa capire come si svolge; il lettore o ascoltatore della narrazione, arrivi a conclusioni simili a quelle tratte dal narratore; le conclusioni scaturiscono dal racconto narrativo; si considera questo racconto una narrazione.
Dalla valutazione dell’articolo di Rosgen et al [20] risulta una congruità tra la metodologia di ricerca e la domanda o gli obiettivi della ricerca; congruità tra la metodologia di ricerca e i metodi utilizzati per raccogliere i dati; congruità tra la metodologia di ricerca e la rappresentazione e l'analisi dei dati; congruità tra la metodologia di ricerca e l'interpretazione dei risultati; i partecipanti e le loro voci sono adeguatamente rappresentati; la ricerca è etica secondo i criteri attuali o, per studi recenti, e vi sono prove di approvazione etica da parte di un organismo appropriato; le conclusioni tratte nel rapporto di ricerca derivano dall'analisi o dall'interpretazione dei dati. Non è stata affrontata l'influenza del ricercatore sulla ricerca e viceversa. Risulta ambigua un'affermazione che colloca il ricercatore culturalmente o teoricamente; la congruenza tra la prospettiva filosofica dichiarata e la metodologia di ricerca.
Dalla valutazione dell’articolo di Speck et al [21] risulta: gli autori sono chiaramente identificati; gli autori hanno esperienza e conoscenza in quel campo di competenza; gli interessi della popolazione coinvolta sono al centro del parere dell’esperto; il parere dimostra un'argomentazione logicamente difesa a sostegno delle conclusioni tratte e c'è un riferimento alla letteratura esistente. Risulta ambigua l’incongruenza con la letteratura/le fonti logicamente difese
In seguito alla valutazione con il JBI's critical appraisal tools 2 articoli sono stati esclusi: l’articolo (14) e l’articolo (23).

Critical Appraisal Tools

**1. Acun et al 2022**	Y (Yes)	N (No)	A (Ambigue)	NA (Not Applicabile)
1. Is it clear in the study what is the "cause" and what is the "effect" (i.e., there is no confusion about which variable comes first)?	X			
2. Were participants included in similar comparisons?		X		
3. Were participants included in comparisons that received a similar treatment/care, other than the exposure or intervention of interest?		X		
4. Was there a control group?		X		
5. Were there multiple outcome measurements both before and after the intervention/exposure?	X			
6. Was the follow-up complete, and if not, were the differences between the groups in terms of follow-up adequately described and analyzed?	X			
7. Were participants' outcomes included in any similarly measured comparisons?			X	
8. Were the results reliably measured?	X			
9. Was an appropriate statistical analysis used?	X			
Overall Rating: **Include**				

2. Andiman et al 2018

		Y (Yes)	N (No)	A (Ambigue)	NA (Not Applicabile)
1.	Is it clear in the study what is the "cause" and what is the "effect" (i.e., there is no confusion about which variable comes first)?	X			
2.	Were participants included in similar comparisons?		X		
3.	Were participants included in comparisons that received a similar treatment/care, other than the exposure or intervention of interest?		X		
4.	Was there a control group?		X		
5.	Were there multiple outcome measurements both before and after the intervention/exposure?	X			
6.	Was the follow-up complete, and if not, were the differences between the groups in terms of follow-up adequately described and analyzed?				X
7.	Were participants' outcomes included in any similarly measured comparisons?	X			
8.	Were the results reliably measured?	X			
9.	Was an appropriate statistical analysis used?	X			
Overall Rating: **Include**					

3. Andreessen et al 2012

		Y (Yes)	N (No)	A (Ambigue)	NA (Not Applicabile)
1.	Is it clear in the study what is the "cause" and what is the "effect" (i.e., there is no confusion about which variable comes first)?	X			
2.	Were participants included in similar comparisons?		X		
3.	Were participants included in comparisons that received a similar treatment/care, other than the exposure or intervention of interest?		X		
4.	Was there a control group?	X			
5.	Were there multiple outcome measurements both before and after the intervention/exposure?	X			
6.	Was the follow-up complete, and if not, were the differences between the groups in terms of follow-up adequately described and analyzed?		X		
7.	Were participants' outcomes included in any similarly measured comparisons?		X		
8.	Were the results reliably measured?	X			
9.	Was an appropriate statistical analysis used?	X			
	Overall Rating: **Include**				

4. **Anthony et al 2011**	Y (Yes)	N (No)	A (Ambigue)	NA (Not Applicabile)
1. Was true randomization used for assigning participants to treatment groups?	X			
2. Has the assignment to treatment groups been hidden?	X			
3. Were the treatment groups similar to baseline?	X			
4. Were the participants blinded to treatment assignment?	X			
5. Were those who provided the treatment blinded to the assignment of treatment?			X	
6. Were the outcome assessors blinded to treatment allocation?			X	
7. Were the treatment groups treated identically apart from the intervention of interest?	X			
8. Was the follow-up complete, and if not, were the differences between the groups in terms of follow-up adequately described and analyzed?	X			
9. Were the participants analyzed in the groups to which they were randomized?	X			
10. Were the results measured in the same way for the treatment groups?	X			
11. Were the results reliably measured?				
12. Was an appropriate statistical analysis used?				
13. Was the study design appropriate and were any deviations from the standard RCT design (individual randomization, parallel groups) taken into account in the conduct and analysis of the study?				
Overall Rating: **Include**				

5. **Baldwin et al 2008**	Y (Yes)	N (No)	A (Ambigue)	NA (Not Applicabile)
1. Have the patient's demographic characteristics been clearly described?	X			
2. Was the patient's medical history clearly described and presented as a timeline?	X			
3. Was the patient's current clinical condition at presentation clearly described?	X			
4. Were diagnostic tests or assessment methods clearly described and the results?			X	
5. Was the intervention or treatment procedures clearly described?	X			
6. Has the post-surgery clinical condition been clearly described?			X	
7. Have any adverse events (harms) or unforeseen events been identified and described?	X			
8. Does the clinical case provide takeaway lessons?	X			
Overall Rating: **Include**				

6. Borgert et al 2017

		Y (Yes)	N (No)	A (Ambigue)	NA (Not Applicabile)
1.	Is the request for revision clearly and explicitly?	X			
2.	Were the inclusion criteria appropriate for the review application?	X			
3.	Was the research strategy appropriate?	X			
4.	Were the sources and resources used to research the studies adequate?	X			
5.	Were the evaluation criteria of the studies appropriate?	X			
6.	Was the critical evaluation conducted by two or more reviewers independently?		X		
7.	Were there any methods to minimize errors in data extraction?		X		
8.	Were the methods used to combine the studies appropriate?		X		
9.	Has the likelihood of publication bias been assessed?		X		
10.	Were the recommendations for policies and/or practices supported by the reported data?	X			
11.	Were the specific guidelines for new research appropriate?	X			
Overall Rating: **Include**					

		Y (Yes)	N (No)	A (Ambigue)	NA (Not Applicabile)
**7.**	**Bruce et al 2015**				
1.	Were the two groups similar and recruited from the same population?	X			
2.	Were exposures measured similarly to assign people to both exposed and unexposed groups?	X			
3.	Has the exposure been measured validly and reliably?		X		
4.	Have any confounders been identified?		X		
5.	Have there been any strategies to address confounders?		X		
6.	Were the groups/participants outcome-unsuccessful at the start of the study (or at the time of exposure)?			X	
7.	Have the results been measured validly and reliably?	X			
8.	Has the follow-up time been reported and sufficient to be long enough for results to occur?			X	
9.	Was the follow-up complete, and if not, were the reasons for the loss of follow-up described and explored?			X	
10.	Have strategies been used to address incomplete follow-up?		X		
11.	Was an appropriate statistical analysis used?	X			
Overall Rating: **Exclude**					

**8.**	**Bullock et al 2017**	Y (Yes)	N (No)	A (Ambigue)	NA (Not Applicabile)
1.	Is it clear in the study what is the "cause" and what is the "effect" (i.e., there is no confusion about which variable comes first)?	X			
2.	Were participants included in similar comparisons?		X		
3.	Were participants included in comparisons that received a similar treatment/care, other than the exposure or intervention of interest?		X		
4.	Was there a control group?			X	
5.	Were there multiple outcome measurements both before and after the intervention/exposure?	X			
6.	Was the follow-up complete, and if not, were the differences between the groups in terms of follow-up adequately described and analyzed?			X	
7.	Were participants' outcomes included in any similarly measured comparisons?			X	
8.	Were the results reliably measured?			X	
9.	Was an appropriate statistical analysis used?	X			

9. Davidson, Enns, Dempster, Lundeen, Eppes 2022

		Y (Yes)	N (No)	A (Ambigue)	NA (Not Applicabile)
1.	Were the two groups similar and recruited from the same population?	X			
2.	Were exposures measured similarly to assign people to both exposed and unexposed groups?	X			
3.	Has the exposure been measured validly and reliably?	X			
4.	Have any confounders been identified?			X	
5.	Have there been any strategies to address confounders?			X	
6.	Were the groups/participants outcome-unsuccessful at the start of the study (or at the time of exposure)?	X			
7.	Have the results been measured validly and reliably?	X			
8.	Has the follow-up time been reported and sufficient to be long enough for results to occur?			X	
9.	Was the follow-up complete, and if not, were the reasons for the loss of follow-up described and explored?			X	
10.	Have strategies been used to address incomplete follow-up?			X	
11.	Was an appropriate statistical analysis used?	X			
Overall Rating: **Include**					

**10.** **Davidson, Enns; Bennet, Sangi-Haghpeykar, Lundeen, Eppes 2022**		Y (Yes)	N (No)	A (Ambigue)	NA (Not Applicabile)
1.	Were the two groups similar and recruited from the same population?	X			
2.	Were exposures measured similarly to assign people to both exposed and unexposed groups?	X			
3.	Has the exposure been measured validly and reliably?	X			
4.	Have any confounders been identified?	X			
5.	Have there been any strategies to address confounders?			X	
6.	Were the groups/participants outcome-unsuccessful at the start of the study (or at the time of exposure)?	X			
7.	Have the results been measured validly and reliably?	X			
8.	Has the follow-up time been reported and sufficient to be long enough for results to occur?	X			
9.	Was the follow-up complete, and if not, were the reasons for the loss of follow-up described and explored?	X			
10.	Have strategies been used to address incomplete follow-up?			X	
11.	Was an appropriate statistical analysis used?	X			
Overall Rating: **Include**					

11. Dieplinger et al 2020

		Y (Yes)	N (No)	A (Ambigue)	NA (Not Applicabile)
1.	Were the two groups similar and recruited from the same population?	X			
2.	Were exposures measured similarly to assign people to both exposed and unexposed groups?	X			
3.	Has the exposure been measured validly and reliably?	X			
4.	Have any confounders been identified?			X	
5.	Have there been any strategies to address confounders?			X	
6.	Were the groups/participants outcome-unsuccessful at the start of the study (or at the time of exposure)?	X			
7.	Have the results been measured validly and reliably?	X			
8.	Has the follow-up time been reported and sufficient to be long enough for results to occur?	X			
9.	Was the follow-up complete, and if not, were the reasons for the loss of follow-up described and explored?	X			
10.	Have strategies been used to address incomplete follow-up?			X	
11.	Was an appropriate statistical analysis used?	X			
Overall Rating: **Include**					

12. Giles et al 2015

		Y (Yes)	N (No)	A (Ambigue)	NA (Not Applicabile)
1.	Is it clear in the study what is the "cause" and what is the "effect" (i.e., there is no confusion about which variable comes first)?	X			
2.	Were participants included in similar comparisons?	X			
3.	Were participants included in comparisons that received a similar treatment/care, other than the exposure or intervention of interest?			X	
4.	Was there a control group?		X		
5.	Were there multiple outcome measurements both before and after the intervention/exposure?	X			
6.	Was the follow-up complete, and if not, were the differences between the groups in terms of follow-up adequately described and analyzed?	X			
7.	Were participants' outcomes included in any similarly measured comparisons?			X	
8.	Were the results reliably measured?	X			
9.	Was an appropriate statistical analysis used?	X			
Overall Rating: **Include**					

13. **Muller et al 2023**

		Y (Yes)	N (No)	A (Ambigue)	NA (Not Applicabile)
1.	Is the source of the opinion clearly identified?	X			
2.	Does the source of the opinion have a position in the field of expertise?	X			
3.	Are the interests of the population concerned at the heart of the opinion?	X			
4.	Does the opinion demonstrate a logically defended argument in support of the conclusions drawn?	X			
5.	Is there a reference to existing literature?	X			
6.	Is there any inconsistency with the logically defended literature/sources?			X	
Overall Rating: **Include**					

14. Resar et al 2012

		Y (Yes)	N (No)	A (Ambigue)	NA (Not Applicabile)
1.	Is the narrative generator a credible or appropriate source?	X			
2.	Is the relationship between the text and its context explained? (where, when, with whom, how)	X			
3.	Does the narrative present events using a logical sequence so that the reader or listener can understand how it unfolds?	X			
4.	Do you, as a reader or listener of the narrative, come to similar conclusions as the narrator?	X			
5.	Do the conclusions come from the narrative story?	X			
6.	Do you consider this story a narrative?	X			
Overall Rating: **Include**					

15. **Rosgen et al 2022**

		Y (Yes)	N (No)	A (Ambigue)	NA (Not Applicabile)
1.	Is there a congruence between the stated philosophical perspective and the research methodology?			X	
2.	Is there a congruity between the research methodology and the research question or objectives?	X			
3.	Is there a congruity between the research methodology and the methods used to collect the data?	X			
4.	Is there a congruity between the research methodology and the representation and analysis of the data?	X			
5.	Is there a congruity between the research methodology and the interpretation of the results?	X			
6.	Is there a statement that places the researcher culturally or theoretically?			X	
7.	Do you deal with the influence of the researcher on the research and vice versa?		X		
8.	Are the participants and their voices adequately represented?	X			
9.	Is the research ethical according to current criteria or, for recent studies, and is there evidence of ethical approval by an appropriate body?	X			
10.	Are the conclusions drawn in the research report derived from the analysis or interpretation of the data?	X			
Overall Rating: **Include**					

16. Speck et al 2016

		Y (Yes)	N (No)	A (Ambigue)	NA (Not Applicabile)
1.	Is the source of the opinion clearly identified?	X			
2.	Does the source of the opinion have a position in the field of expertise?	X			
3.	Are the interests of the population concerned at the heart of the opinion?	X			
4.	Does the opinion demonstrate a logically defended argument in support of the conclusions drawn?	X			
5.	Is there a reference to existing literature?	X			
6.	Is there any inconsistency with the logically defended literature/sources?			X	
Overall Rating: **Include**					
